# Supplementary material for: An open label, block randomized, community study of the safety and efficacy of co-administered ivermectin, diethylcarbamazine plus albendazole vs. diethylcarbamazine plus albendazole for lymphatic filariasis in India
Source: PLoS Negl Trop Dis. 2021 Feb 16;15(2):e0009069. doi: 10.1371/journal.pntd.0009069 (PMC7909694; doi:10.1371/journal.pntd.0009069)
Supplement: S1 Protocol — (PDF) [file pntd.0009069.s001.pdf]

A community based study, to compare the safety, efficacy and acceptability of a triple drug regimen (Ivermectin, Diethylcarbamazine and Albendazole) with a two-drug regimen (Diethylcarbamazine and Albendazole) for lymphatic filariasis elimination programme

Vector Control Research Centre
(Indian Council of Medical Research)
Indira Nagar
Puducherry – 605006, India

In collaboration with

National Vector Borne Disease Control Programme (NVBDCP)
Delhi and Karnataka

Duration of the study
July 2016 – March 2018

Funding agency

Neglected Tropical Diseases Support Center (NTD-SC)
Task Force for Global Health
325 Swanton Way, Decatur GA 30030
Tel: 404.687.5621, Fax: 404.371.1138

(Revised on 20 September 2017 for Phase II study – effectiveness and efficacy)

INVESTIGATORS

Principal Investigator

1. Dr. P. Jambulingam
   Scientist "G" and Director
   Vector Control Research Centre
   Pondicherry

Co-Principal Investigator

2. Dr. Vijesh Sreedhar Kuttiatt
   Scientist "E"
   Vector Control Research Centre
   Pondicherry

Co-Investigators

3. Dr. R. J. L. De Britto
   Scientist "F"
   Vector Control Research Centre
   Pondicherry

4. Dr. S. Subramanian
   Scientist "F"
   Vector Control Research Centre
   Pondicherry

5. Ms. A. Srividya
   Scientist "D"
   Vector Control Research Centre
   Pondicherry

6. Ms. B. Nandha
   Scientist "B"
   Vector Control Research Centre
   Pondicherry

7. Dr. K. Krishnamoorthy
   Consultant (Rtd. Scientist "G")
   Vector Control Research Centre
   Pondicherry

Project Co-ordination: Indian Council of Medical Research, New Delhi

Collaboration: National Vector Borne Disease Control Programme (NVBDCP)
Delhi and Karnataka

Table of contents

Page No.

LIST OF ABBREVIATIONS 5

1. **PROTOCOL SUMMARY** 6
2. **BACKGROUND AND RATIONALE** 8
3. **OBJECTIVES** 9
4. **STUDY DESIGN** 9
   - **4.1 Selection of study sites** 10
   - **4.2 Preparatory activities** 11
   - **4.3 Pre-treatment assessments** 12
   - **4.4 Drug administration** 13
   - **4.5 Rapid Response for management of adverse events** 13
   - **4.6 Early post-treatment assessment** 13
   - **4.7 Data safety review board (DSRB)** 15
   - **4.8 Acceptability of triple drug regimen** 15
   - **4.9 One year post-MDA assessment** 16
   - **4.10 Treatment** 17
   - **4.11 Data Collection** 17
5. **STATISTICAL CONSIDERATIONS** 18
   - **5.1 Safety data** 18
   - **5.2 Effectiveness** 18
   - **5.3 Efficacy** 18
6. **BIOHAZARD CONTAINMENT** 19
7. **DISSEMINATION ACTIVITIES** 19
8. **TEAM COMPOSITION AND WORK PLAN** 19
9. **TIMELINE OF ACTIVITIES** 22
10. **BUDGET** 23
11. **REFERENCES** 24

Table 1 Mf prevalence in sentinel and spot check sites in Yadgir district in 2014 and 2015 25

Table 2 Drug dosage for DA arm (as per national Guidelines) 26

Table 3 Weight based dosage for ivermectin (all persons of age > 5 years with weight ≥ 15 Kg) .. 27

Table 4 Drug requirements for the two arms, IDA and DA 28

Part A - Information sheet 61

Part B **(adults/ adolescents)** 64

Part C – Assent Form for children in the age class 7-17 years 65

Part D - Parental Informed Consent form 66

Appendix 1a	Thomsen *et al (2015) Clinical Infectious Disease*
Appendix 1b	Gyapong *et al* (2005) *Expert Opinion Pharmacotherapy*
Appendix 2	Background on study drugs
Appendix 3	Study flow
Appendix 4	Information sheet and consent form
Appendix 5	Participant enrolment form
Appendix 6	Participant monitoring form
Appendix 6a	Guide for assigning adverse event severity grades 1-4
Appendix 7	Adverse event evaluation and report form
Appendix 7a	Instructions for reporting severe adverse events
Appendix 8	Medical monitoring activities
Appendix 9	Treatment acceptability study guidelines

LIST OF ABBREVIATIONS

Ab	Antibody
AE	Adverse Event/Adverse Experience
AEERF	Adverse Event Evaluation and Report Form
Ag	Antigenemia
ALB	Albendazole
CRF/eCRF	Case Report Form also referred to as eCRF (electronic Case Report Form)
CRO	Contract Research Organization
DA	Diethylcarbamazine and Albendazole (Two Drug Therapy)
DEC	Diethylcarbamazine
DOT	Directly Observed Treatment
DSRB	Data Safety Review Board
IHEC	Institutional Human Ethics Committee
EDC	Electronic Data Capture
FTS	Filariasis Test Strip
GPELF	Global Programme to Eliminate Lymphatic Filariasis
GPS	Global Positioning System
ICF	Informed Consent Form
ICMR	Indian Council of Medical Research
IDA	Ivermectin, Diethylcarbamazine and Albendazole (Triple Drug Therapy)
IVM	Ivermectin
KAP	Knowledge, Attitude and Practices
LF	Lymphatic Filariasis
MDA	Mass Drug Administration
Mf	Microfilaria(e)
NTD	Neglected Tropical Diseases
SAE	Serious Adverse Event/Experience
SOP	Standard Operating Procedure
TAS	Transmission Assessment Survey
VCRC	Vector Control Research Centre
WHO	World Health Organization

1. PROTOCOL SUMMARY

**Study Title**	A community based study to compare the safety, efficacy and acceptability of a triple drug regimen (Ivermectin, Diethylcarbamazine and Albendazole) with a two-drug regimen (Diethylcarbamazine and Albendazole) for lymphatic filariasis elimination programme
**Type of Study**	Community based open intervention study
**Population**	IDA/ Triple Drug Arm: participants more than or equal to 5 years of age and above 15 Kg body weight<br><br>DA/ Dual Drug Arm (DA): participants more than or equal to 2 years of age
**Number of Treated Areas**	Selected communities in Yadgir district, Karnataka based on the results of Mf survey in sentinel and spot-check sites
**Duration of participation of community members**	Single treatment and daily monitoring of adverse events through Day 7<br>Follow up for infection at 1 year post treatment.
**Study Drugs**	Arm 1 (Co-administration of three drugs)<br>Ivermectin (3 mg tablets) - 200 µg /kg<br>Diethylcarbamazine (100 mg tablets) - 6mg/kg<br>Albendazole (400 mg tablets) - flat dose of 400 mg<br>Arm2 (Co-administration of two drugs)<br>Diethylcarbamazine (100 mg tablets) - 6mg/kg<br>Albendazole (400 mg tablets) - flat dose of 400 mg
**Objectives**	• To determine the frequency, type and severity of adverse events following triple-drug therapy (IVM+DEC+ALB, IDA) compared to the standard two-drug treatment (DEC+ALB, DA) in infected and uninfected individuals in a community.<br>• To compare the efficacy of IDA vs. DA administered in communities for clearance of Mf and filarial antigenemia (Ag) in cohort and effectiveness (prevalence) in community settings.<br>• To assess and compare the prevalence of antibody (Ab) with that of Ag and Mf<br>• To assess the presence and intensity of filarial infection on the frequency and severity of adverse events.<br>• To compare community acceptance of MDA with IDA vs. DA.

General flow diagram

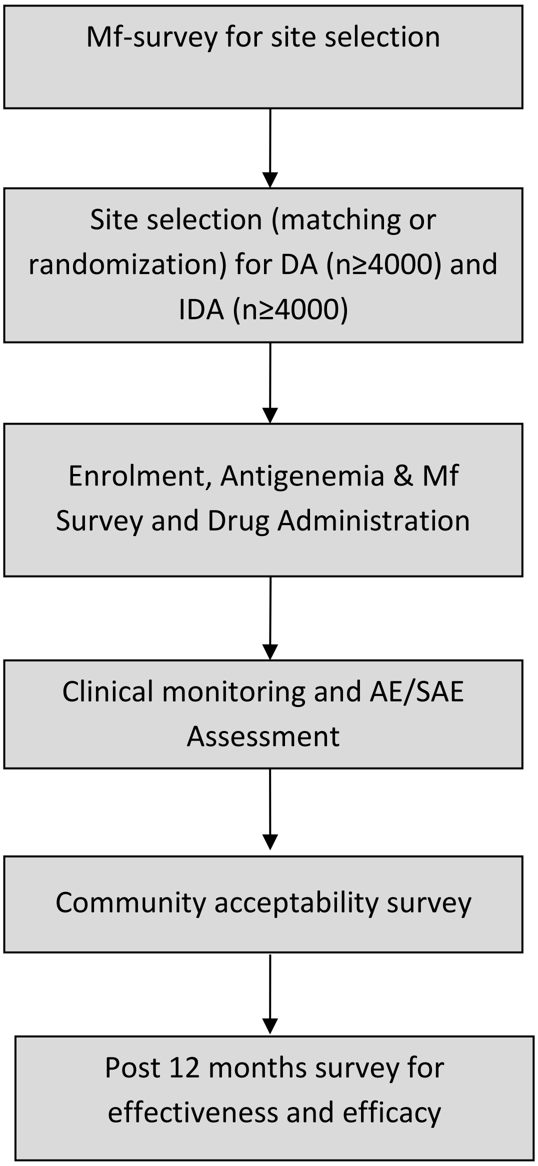

2. BACKGROUND AND RATIONALE

In 2000, WHO launched the Global Programme to Eliminate Lymphatic Filariasis (GPELF) to eliminate lymphatic filariasis as a public health problem by 2020[1]. The National Health Policy of India2002 set the goal of achieving elimination of lymphatic filariasis (LF) in the country by 2015. The National Vector-Borne Disease Control Programme (NVBDCP) launched the programme for elimination of LF (PELF) in 2004 and adopted the WHO recommended two-drug policy of co-administration of diethylcarbamazine (DEC) and albendazole in 2007 [2]. The programme's current strategy to interrupt transmission relies on annual single dose mass drug administration (MDA) of DEC and albendazole (DA) given to the eligible population in endemic districts [3]. Disability alleviation and morbidity prevention activities include home based management of lymphoedema and surgery for hydrocele.

The programme has made significant progress with Mf levels below 1% in 222 of 255 implementation units (IUs) in 2015. Fifty-three IUs have already cleared TAS-1 while 65 IUs will conduct TAS1 and another 4 IUs are planning TAS2. Next year's round of MDA will target133 IUs. Despite this success in the majority of districts, microfilaria (Mf) levels have remained >1% in 31 "hard-core" foci (districts). The programme has been looking for additional tools to accelerate interruption of transmission in these districts and ensure that it meets the goal of elimination by 2020.

Results of a pilot study [4] (**Appendix 1a**) now confirmed with results from a clinical trial in Papua New Guinea (PNG) showed that triple drug therapy [ivermectin, DEC, albendazole (IDA)] is superior to the currently recommended two-drug regimen [5] (**Appendix 1b**) used in the global programme to eliminate LF (GPELF) outside of sub-Saharan Africa (DEC, albendazole [DA]). A single dose of IDA rapidly achieved complete clearance of *Wuchereria bancrofti* microfilariae from the blood of 12 individuals for at least one year post-treatment. All six individuals tested at 24 months were still amicrofilaraemic at that time. These results suggest that IDA permanently sterilizes adult filarial worms. Many people treated in these studies experienced transient systemic adverse events (AE) commonly associated with DEC or ivermectin treatment of filariasis, and AEs were more frequent after IDA than after DA. However, no serious adverse events (SAE) were observed in these trials or in a trial that is currently in progress in the West African country of Côte d'Ivoire. No information is available on the frequency or type of AEs following IDA treatment of uninfected persons, but this is expected to be low. The dramatic reduction and sustained decrease of mf (zero at 1 year) along with the safety profile seen in the PNG studies suggest that the triple drug therapy may be a useful tool for eliminating LF in districts in India where Mf rates have remained > 1% following MDA with the standard DA regimen. Ivermectin also provides additional benefits for recipients, because it complements the

deworming effect of albendazole and because of its effect against lice and scabies and mites. Pharmacokinetic studies done in PNG indicated no significant effect of ivermectin on DEC or albendazole drug levels [4] (**Appendix 1a**).

IDA's potential to accelerate LF elimination in India and around the world has stimulated WHO's interest as well as academic experts and the donor community. Although the studies cited above have clearly demonstrated the superiority of the IDA regimen for clearing *W. bancrofti* mf from the blood, more safety and efficacy data are needed before IDA can be rolled out on a large scale as an MDA regimen for India. WHO recommends a best practice called "cohort event monitoring" for demonstrating safety of new drug regimens for public health program use. Establishing safety for MDA through such methodology requires pre and post treatment assessment from at least 10,000 people treated across multiple settings (minimum 4000 from India). The current two-drug MDA regimens were studied in closely monitored community trials in a similar manner before they were endorsed for widespread use in the GPELF. An expanded discussion of mechanisms of action and side effects of ivermectin, DEC and albendazole is provided in **Appendix 2**. It is therefore proposed to conduct a study to acquire similar safety data in India before the new IDA regimen can be used in those districts where Mf levels have remained > 1% with the following objectives:

3. OBJECTIVES

i. To determine the frequency, type and severity of adverse events following triple-drug therapy (IVM+DEC+ALB, IDA) compared to the standard two-drug treatment (DEC+ALB, DA) in infected and uninfected individuals in a community.
ii. To compare the efficacy of IDA vs. DA administered in communities for clearance of Mf and filarial antigenemia (Ag) in cohort and effectiveness (prevalence) in community settings.
iii. To assess and compare the prevalence of antibody (Ab) with that of Ag and Mf
iv. To assess the presence and intensity of filarial infection on the frequency and severity of adverse events.
v. To compare community acceptance of MDA with IDA vs. DA.

4. STUDY DESIGN

This trial will be an open labelled two-armed study. The two arms are (1) MDA with IDA (triple drug therapy) and (2) MDA with the currently used combination of DA (two-drug regimen). An overview of the study design is provided in **Appendix 3**.

4.1 Selection of study sites

The study will be conducted in Yadgir district (carved out of Gulbarga district) in Karnataka state (Map), where 12 rounds of MDA have been conducted since 2004. Mf prevalence has been shown to be persistently above 1% during annual surveys conducted by the programme. Impact assessment conducted in 2014 and 2015 (prior to 11 and 12th round of MDA) in the sentinel and spot check sites (villages) have shown many sites with >1% Mf prevalence **(Table 1)**. As the last MDA took place in Dec 2015 after the impact assessment, it is necessary to assess current prevalence of microfilaraemia to select study sites for the present study. The Mf survey will be carried out jointly by the research team and State NVBDCP as a part of impact assessment of the programme in sentinel sites as well as spot-check sites.

Map. Sites showing >1% Mf prevalence in Yadgir district, Karnataka in 2014

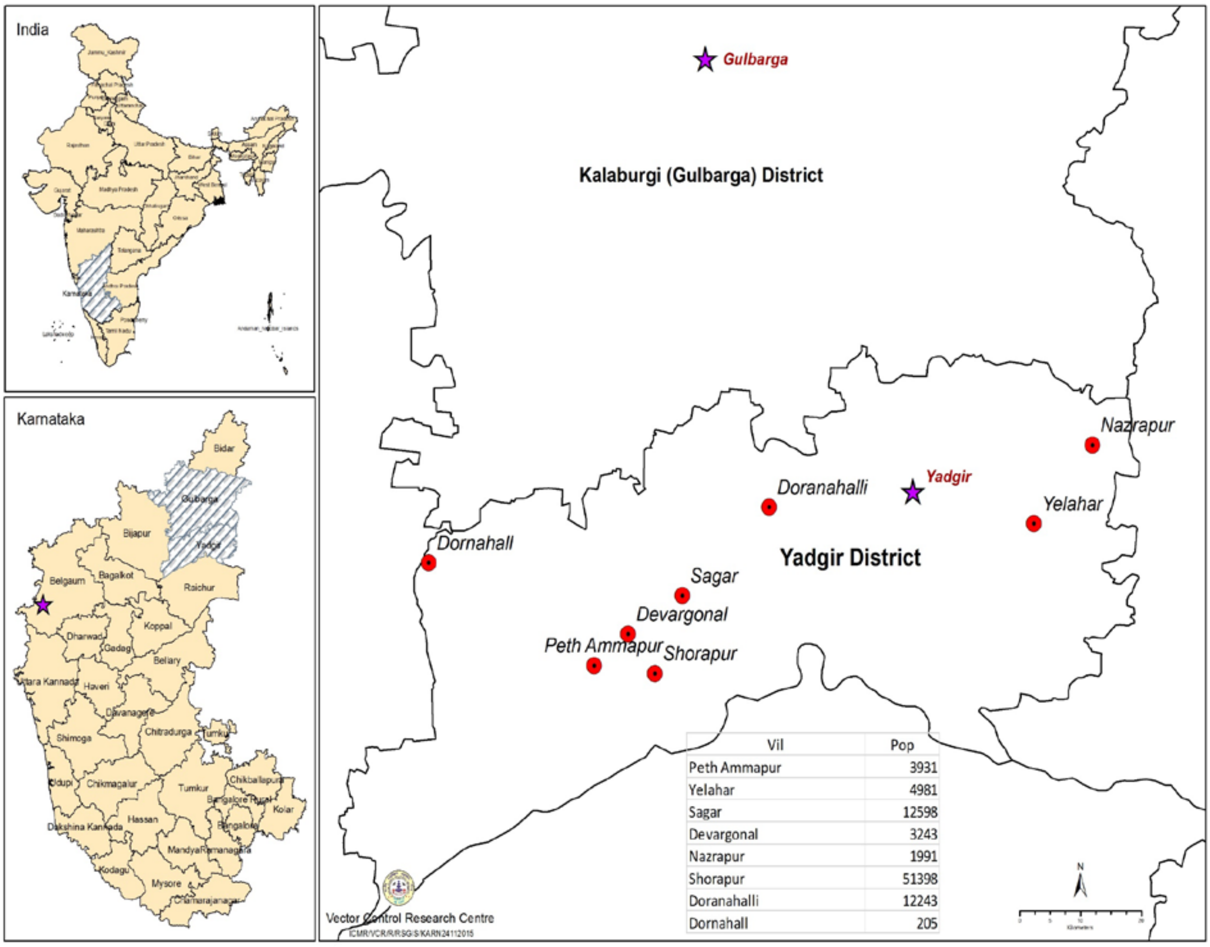

The minimum sample size required is 550 for a community with a population of 2000. The sample size is calculated based on an Mf prevalence of 2% with an error margin of 1% (expected prevalence is in the range of 1-3%) and 95% confidence level. Accordingly, a sample of 600 persons will be selected from each community (sentinel/ spot check sites) with a known prevalence of >2% and examined for

Mf. For this purpose, 60 µl blood will be sampled from each selected and consenting person. This way, the sites selected for the study are expected to have prevalence of at least 1% during the participant enrolment survey (pre-treatment assessment). In case, if the Mf-prevalence is less than 1% after pre-treatment assessment, additional sites will be selected based on risk ranking (line listing of LF cases) to augment the required minimum sample of 4000 in each arm.

Based on the results of the survey, the study sites will be selected and grouped into two arms of 6000 population each with comparable Mf prevalence. Sites will be assigned for MDA either by randomization or by purposive matching considering the population and prevalence of Mf. If the prevalence is homogenous across the sites, each site will be randomly assigned to one of the two treatment arms. If the prevalence is heterogeneous, sites will be selected into each arm so that the population and prevalence between the two treatment arms are similar.

4.2 Preparatory activities

4.2.1 Social mobilization

Prior to the administration of the drugs intense social mobilization activities (**Home Visit #1, Appendix 3**) will be conducted to ensure maximum community participation. Briefly, this will include development and distribution of key messages that will emphasize the acceptance and swallowing of the drugs, along with their benefits and safety. Print and electronic mass media, people-based and folk media will be used in this campaign.

4.2.2 Participant enrolment

Teams will enumerate and record the GPS coordinates of each house within the selected sites **(Home Visit #2, Appendix 3)**. Prior to enumeration, all eligible individuals will sign a written, informed consent **(Appendix 4)**. In the event that a subject is unable to read or has insufficient level of knowledge to comprehend the consent form, another villager with sufficient reading and writing skills will act a witness to the consenting process. At this time individuals will be evaluated as to whether they fulfil the inclusion/exclusion criteria, before they give informed consent. Each individual in the household will be assigned a unique barcode ID and their personal details (name, age and sex) will be entered in an enrolment e-form (**Appendix 5**). Household level information on presence of screened windows, and use of bed net will also be collected. Eligible individuals for MDA will be identified and included in the study.

4.2.3 Eligibility

All participants will provide written informed consent before any study procedures are done. Participation of minors (less than 18 years of age) will require their assent and the written consent of at least one parent.

Inclusion criteria

(i) Age ≥ 5 years and body weight above or equal to 15 Kg, male or female for IDA area and age ≥2 years for DA arm.

(ii) Able to provide informed consent or give parental consent to minors to participate in the study

(iii) No evidence of severe or systemic co-morbidities except for features of filarial disease

Exclusion criteria

Participants are ineligible to participate in the study, if they have any of the following:

(i) Age < 5 years (ivermectin is not approved for use in children less than 5 years of age) and age 5 years and above with body weight below 15 Kg for IDA arm and age < 2 years for DA arm

(ii) Pregnant women (***DEC, ivermectin and albendazole are not known to be safe for use during pregnancy***) and women of child bearing age who cannot recall the timing of their last menstrual period or who report that their last menstrual period started 4 weeks or longer before the enrolment.

(iii) Severe chronic illness (for example, chronic renal insufficiency, severe chronic liver disease or any illness that is severe enough to interfere with activities of daily living)

(iv) History of previous allergy to MDA drugs

4.3 Pre-treatment assessments

4.3.1 Health assessment

Each individual will be questioned whether they have a history or signs of LF (hydrocele, lymphedema, lymphangitis, lymphadenitis) and whether they had previous MDA for LF and the responses will be recorded in the enrolment form **(Appendix 5)**. They will also be asked whether they recently had taken albendazole or ivermectin for other conditions. If participants have any potential physical findings associated with LF they will be examined for those conditions. The study population will also have the same AE evaluation that will be used following treatment. This will include a questionnaire of subjective finding such as headache, joint pain, etc. and presence of scabies. This will establish a baseline for later assessment of AEs. Individuals will also be questioned for general health (especially

acute illness or serious chronic illness) and on last menstrual period to women participants (to establish pregnancy for women of childbearing age).

4.3.2 Screening for filarial antigenemia and microfilaraemia

All the eligible individuals in the selected community ≥5 years of age will be screened using WHO approved point-of care filarial antigen test (with approximately 75 µl blood by finger prick), and those with positive antigen test will be visited at night (8- 11 pm) for microfilaria testing (60µl measured volume thick blood smear) by finger prick method. Blood collection via finger prick is considered to be minimal risk and little or no discomfort is anticipated. The results of the tests will be recorded in the enrolment e-form **(Appendix 5).**

4.4 Drug administration

The two drug combination is a standard MDA with DEC (6mg/kg) and albendazole (flat dose of 400 mg). The triple drug administration will consist of a single dose of ivermectin (200 µg /kg) added to the standard MDA. The dosage schedule for the two drug standard MDA is given in **Table 2** and ivermectin will be based on body weight **(Table 3)**. Study personnel will directly administer the drugs. The study population will be encouraged to have food before swallowing the medicine (without chewing the tablets) with a glass of water. Vomited doses will be replaced. The study population will be informed about the active follow-ups for adverse events at 12h and 48h, and for passive evaluation up to day 7 following treatment. The drugs (quality assured) will be supplied by the companies donating for LF elimination programme and the requirement is shown in **Table 4**.

4.5 Rapid Response for management of adverse events

Medical teams will be located at strategic places. People and the drug administrators will be informed about the availability of such teams including the mobile phone numbers so that they can report directly to these teams, if necessary. These teams will be in position from the day of drug administration until the completion of mopping up operations. Each team will have an ambulance with a medical officer, a staff nurse and a pharmacist and essential life-saving drugs.

4.6 Early post-treatment assessment

4.6.1 Tiered Adverse Events Monitoring and Management

DEC, ivermectin and albendazole are known to produce adverse events in some treated persons and are self-limiting. These events can be non-specific drug related reactions which include headache,

anorexia, nausea, abdominal pain, vomiting, dizziness, weakness or lethargy. These symptoms begin within 1-2 hours of taking the drug and persist for a few hours and may disappear spontaneously with or without symptomatic treatment. Specific parasite related allergic reactions due to destruction of microfilariae and adult worms include fever, local inflammations around dead worms and pruritus.

The team that administered the drugs will visit the “treated households” for active monitoring of adverse events approximately 12 and 48 hours following drug administration. Every treated person in the community will be actively sought to have two post-treatment active event-monitoring sessions. Most adverse events, especially the more severe, occur in the first 12 - 48h following treatment associated with killing of microfilaria. However, occasional adverse events related to adult worm death may be delayed by several days. Formal assessment of adverse events (with a standard form) will take place on days 1 and 2 and later if symptoms persist or start late. To capture these late adverse events and to assure that any systemic adverse events that occurred earlier have resolved, the team will also visit study villages daily on days 3 through 7 after treatment to record any report of persistent or late-developing AEs. These evaluations will be documented by filling in pre-printed monitoring forms **(Appendix 6)** using the scoring instructions for AEs **(Appendix 6a)** and directly entered data into tablet computers using a program such as Redcap or EpiInfo.

Mild symptoms and management: In the case of mild symptomatic reactions (objective and localized: fever, lymphadenitis, scrotal pain, scrotal swelling, proteinuria, haematuria; subjective or systemic: headache, light headedness, nausea, vomiting, abdominal pain, joint pain, malaise, dizziness) local health workers/ study team will give antipyretics/analgesics and anti-allergic agents at the time of follow-up.

Moderate symptoms and management: Individuals with AEs that interfere with work or school will have more detailed assessments with a brief physical examination with measurement of temperature, blood pressure and pulse by the physician. If the initial AE monitoring reveals AEs that (a) interfere with daily activities and/or a temperature >39^0C (b) a significant drop in blood pressure and (c) other significant objective findings they will be evaluated for potential severe adverse event. The physician will provide any required immediate treatment and facilitate admission into the hospital or health centre, if deemed necessary. All events with grades ≥ 3 or overnight hospitalization will be referred as Severe Adverse Events that require completion of the Adverse Event Evaluation and Report Form (AEERF, **Appendix 7**).

4.6.2 Severe adverse event assessment and management protocol

The study population with definite or suspected severe AEs will be referred to medical personnel for further evaluation. These evaluations will be documented with AEERF forms **(Appendix 7)**, following the instructions **(Appendix 7a)**. An independent Medical Monitor who will decide whether the AEs reported are related, possibly related, or unrelated to the treatment will review all severe adverse event assessment evaluations. Severe adverse event assessment reports will be sent electronically to the Independent Medical Monitor (who has received GCP training and is experienced in clinical trials and management of AEs following treatment of lymphatic filariasis). Classification of events as Serious Adverse Events and causation (definitely related to MDA, probably, possible, or unrelated) will be based on the attending physician’s report and the medical monitor’s opinion. Medical monitor will forward severe adverse event assessment reports to the data safety review board. Individuals with severe adverse events (whether related or unrelated to drug treatment) will be hospitalized if necessary and followed until their symptoms have resolved. All severe adverse events will be reported within 48 hours. The report will be submitted to the Chairperson of DSRB nominated by Govt. of India within 7 days and the Institutional Humans Ethics Committee (IHEC). Server Adverse event is considered as serious adverse event/experience (SAE) when the severity grade is 4 and above.

4.6.3 Compensation for Injury

The study drugs have been widely used for treatment of lymphatic filariasis and it is anticipated that injury resulting from treatment will be rare. In the event that a participant experiences severe adverse event attributable to study treatment, expenses towards medical treatment and/or hospitalization will be covered through medical insurance. Participants reporting with severe adverse events in the grade 5 will be covered under liability insurance policy.

4.7 Data safety review board (DSRB)

The DSRB will monitor the type and frequency of AEs and SAEs recorded by the teams and provide guidance to the teams in the field. The DSRB and the Institutional Humans Ethics Committee (IHEC) may conduct a review after MDA was distributed to 500 people in each treatment area and to consider stopping the study if 5 or more of 500 people experienced SAEs attributable to MDA.

4.8 Acceptability of triple drug regimen

Community acceptance will be measured by surveying community members receiving either the 2-drug or 3-drug regimens. A survey questionnaire will be designed, pre-tested and used to assess what

respondents know about LF, what they think of people with LF, how do they perceive LF affects their health, how the health system manages people with LF, especially their perceptions on study drug administration and of reactions to treatment and fears of being treated. The sample will include two categories of individuals: (i) Mf and Ag positive and (ii) Mf and Ag negative. In estimating the sample size for the acceptability survey, estimated acceptability rates are available only for 2-drug regimen. In the absence of such information for 3-drug acceptability rate, the difference in the estimate that may be expected between the regimen groups cannot be derived for estimating the sample size. As a result, this survey will create preliminary data, providing an insight into possible trends in acceptability. Therefore, a minimum of 100 individuals above 14 years will be randomly selected in each category for the survey in each arm. All the Mf and Ag positive individuals (above 14 years) will be included if the number is below 100.

To complement this survey, a series of focus group discussions (FGD) in the community as well as key informant interviews (community leaders, health personnel and drug distributors in the same communities) to assess perceptions about the 3-drug versus the 2-drug regimen will be conducted at the same time along with community survey. Persons from specific groups of people such as women of reproductive age, young people, men and community health workers will be included in the FGD to understand their perspectives on DOT, AE and messaging for the 3-drug regimen. A purposive sampling frame will be used, with individuals identified based on their leadership and cultural position with the village as well as their involvement with LF elimination and with the community trial. A range of 8-10 individuals will be included from each arm.

Guidelines for developing questionnaire for community acceptability survey, Key informant interview and focus group discussion are given in **Appendix 8**.

4.9 One year post-MDA assessment

4.9.1 Effectiveness of IDA versus DA

Effectiveness will be assessed by comparing the pre and 12 months post-MDA prevalence of Mf and Ag, and intensity of Mf between the two arms, by testing a cross section of the population in each arm. Finger prick blood sample measuring 75 µl, and six blood spots (120 µl), each with 20 µl blood on a filter paper for detecting Ag and antibody (Ab) respectively will be collected from the consenting individuals in the selected households between 1700 and 2100 hrs by door to door visit. FTS will be used for detecting Ag and Wb123 or Bm14 will be used for Ab. All Ag-positives will be further blood

smeared for Mf between 2100 and 2300 hrs on the same day. All samples will be processed following respective SOPs for FTS, antibody and Mf.

4.9.2 Efficacy of IDA versus DA

Foe assessing efficacy, individuals who were positive for either Mf or Ag, prior to MDA will be re-examined for Ag. Only those positive for Ag will be further examined for Mf. Majority of positive cohorts (Ag and Mf) are expected to be covered under the effectiveness survey. The remaining positive cohorts will be tested separately by visiting their households.

4.10 Treatment

Person positive for Ag or Mf will be treated with standard MDA regimen (single dose of DEC with albendazole).

4.11 Data Collection

4.11.1 Pre-treatment

Data will be collected using tablet-based systems, which will be pre-loaded with appropriate software. Field teams will be trained in the use of the instruments and data will be uploaded as soon as entries are completed. The secondary data available on other characteristics of the population such as migratory pattern, prevalence of co-morbid conditions and malnutrition status will be collected. Information on the vectors and transmission parameters will be collected.

4.11.2 Post-treatment

The 12-month post-MDA survey for both efficacy and effectiveness assessment will carried out simultaneously. For the effectiveness study, a database developed in EPIINFO software (CDC, Atlanta) will be used to capture data using Android based mobile phones. The data for the cohort will also be captured using tablet-based electronic data capturing (EDC) system that was developed and used during pre-MDA survey. The details of all individuals in the cohort will be pre-loaded and used to capture data during the 12-month post-MDA survey.

5. STATISTICAL CONSIDERATIONS

5.1 Safety data

The sample size of ≥4000 in each arm is sufficient to test the hypothesis that the rates of severe AEs following IDA or DA are less than 0.1%. It is well known that systemic AEs are related to killing of Mf and that the severity of AEs is related to Mf counts. Since Mf rates in villages in the study area are relatively low, the study will not be powered to compare rates of SAEs by MDA regimen. The primary endpoint for safety studies will be the rates of SAEs that occur in infected and in uninfected subjects within the first 7 days post MDA.

5.2 Effectiveness

With a pre-MDA Mf prevalence of ~6% in each arm, the Mf prevalence is expected to be ~2% 12-months post-MDA considering a coverage of 70% under MDA. Assuming both IDA and DA are equivalent ('equivalence trial') in clearing infection and that a difference of 1.2% in infection prevalence is considered as equivalent, a sample of 2137 individuals per arm is sufficient to compare the effectiveness between the arms. This sample size has 80% power of detecting the true difference of more than 1.2% and no more than 5% chance of falsely concluding that the difference is less than 1.2% when in fact it is more than 1.2%. Since household is the sampling unit, a design effect of 1.5 was applied and the target sample is 3205 per arm. Further, the sample size was adjusted to account for a non-response rate of 10%, implying that ~3600 individuals to be contacted to achieve the sample size per arm. With an average family size of 5, a total of 720 households per arm need to be selected following systematic sampling method. All the consenting individuals aged five and above will be tested for filarial infection in both the arms.

5.3 Efficacy

Assuming an Mf-prevalence of 1% in the study population at baseline, the survey is expected to detect at least 60 Mf positives in each arm. A minimum of 35 of these Mf-positives in each arm will be retested at 12 months post-treatment for antigenemia and microfilaraemia. This sample size is adequate to demonstrate superiority of the IDA regimen (assumptions: 90% reduction in Mf-prevalence after IDA and 60% reduction after DA, 80% power for detecting an effect size of 30%). The primary endpoint for efficacy will be complete clearance of Mf 12 months post MDA. Another secondary endpoint will be clearance of filarial antigenemia at 12 months post MDA.

5.4 Follow-up of pre-MDA positives

From human ethics point of view, all the Ag positive (1263 in IDA and 1032 in DA) and Mf positive (311 in IDA and 265 in DA) individuals detected during enrolment are required to be retested for the current status of infection and retreated if they continue to show positive after single dose of treatment in the respective arm. Therefore, the Ag and Mf positives who are not covered under effectiveness survey will be approached and if available, they will be tested for Ag. Ag positive individuals will be further tested for Mf.

6. BIOHAZARD CONTAINMENT

Universal precautions for people collecting blood and working with blood samples and proper disposal of contaminated materials (test strips, lancets, capillary tubes, blood film slides) will be adhered in accordance with the guidelines prescribed by the local health authorities.

7. DISSEMINATION ACTIVITIES

A dissemination workshop will be held after the results are available to inform other stakeholders. The results will be published in an open access peer reviewed journal.

8. TEAM COMPOSITION AND WORK PLAN

(i) A Social Scientist assisted by a Junior Social Worker, PHC level health educator and community liaison persons will be involved in each arm to carry out social mobilization activities in the preparatory phase of the study. This activity will precede participant enrolment. The sociologist team will approach the households, carry out campaigns and prepare the community for the MDA. Each team is expected to complete 140 houses every week. The sociology teams will also be involved in assessing the acceptability using community survey, FGD and KI interviews after the completion of drug administration within the recall period of one month.

(ii) Another team of six members (one physician, two laboratory technicians one each for sample collection and reading antigen test results, one junior nurse, one data entry operator and one field assistant) will be responsible for the following activities: consenting, enumeration, pre-treatment assessments, drug administration and post-treatment AE assessments. The drug distributor in the routine MDA programme in the villages and/or community liaison persons will be a part of the team. There will be 7 such teams in each arm of the study. Each team will visit

the house, get the consent from the members, assess pre-treatment health status, screen all the available individuals for filarial antigenemia and administer drugs except those who are positive for antigenemia. Antigen positive individuals will be tested for microfilaraemia by collecting night blood sample after 9.00 PM and then treated.

(iii) A team is expected to complete 8-10 households (~40 individuals) in a day. During the first door to door visit, the team will enrol the participants, assess health parameters, screen the individuals for filarial infection and provide directly observed treatment. The team will revisit the "treated households" for active monitoring of adverse events if any, to manage the AEs on day 1 and 2 post treatment. Once these activities are completed in these houses, the team will move on to the next batch of 10 houses. A team is expected to cover 16-20 households in a week. All the data pertaining to these activities will be entered in the e-formats by the DEO in the field itself. In this process, a minimum 8-10 weeks is necessary to cover the target population in both the arms (refer section 9). A team with similar composition will be formed for assessing Ag and Mf prevalence after 12 months post treatment and to treat the positive cases.

(iv) A separate team of a physician and a junior nurse will passively monitor the treated individuals (20 - 30 households in a day) for an extended period of 7 days following the treatment in each arm. This team will be supported by the existing Rapid Response Team (RRT) in the District monitoring and managing the AEs in both the arms.

(v) The Medical Officer(s) and Health Inspectors in the PHCs of respective arms will assist the investigators in co-ordinating the activities of the team in the villages and referrals. At the District level, the District Health officer (DHO) and the District Vector Borne Disease Control Officer (DVBDCO) will provide assistance in selection and organisation of teams for various activities including the PHC staff and conducting training programmes and overall supervision. The District Health officer will provide support in terms of deploying the Rapid Response Team, referral arrangements and Hospital support.

(vi) There will be an independent Medical Monitor who will receive severe adverse event assessment reports electronically from the attending physician and forward the report with opinion to the DSRB through the PI/Co-PI.

(vii) All the members of the teams who have basic medical skills will be trained on AE evaluations. Laboratory Technicians will be trained on blood sampling, smearing, processing and examination

of slides for Mf, and reading of antigen test results. Hands on training will be given to the data entry operators on geo-referencing the households and filling up of e-forms using e-tablets pre-loaded with appropriate software.

9. TIMELINE OF ACTIVITIES

Activities and time line

S.No.	Activity	Target	No. of Teams	No. of person / team	Max Days	Jul-16				Aug-16				Sep-16				Oct-16				Nov-16				Dec 2016 - Nov 2017	Oct-17	Nov-17	Dec-17	Jan-18	Feb-18	Mar-18
						W1	W2	W3	W4	W1	W2	W3	W4	W1	W2	W3	W4	W1	W2	W3	W4	W1	W2	W3	W4		W1-W4	W1-W4	W1-W4	W1-W4	W1-W4	W1-W4
$1	Staff Recruitment and training				20	Recruitment																					Recruitment					
$2	Pre screening for Mf for site selection	4000	8	3	12			DHO	DHO																							
$3	Selection of study sites and advocay																															
$4	Preparation of IEC and training the field teams																															
$5	Social mobilization for screening and drug distribution	2400 HHS	2	2	66							B	B	B	B	B	B	B	B	B	B	B	B									
$6	Sensitization and training for Rapid Response team				7						DHO																					
$7	1. Patient enrolment<br>(i) Obtaining consent<br>(ii) Pre-MDA health assessment<br>(iii) Screening - Ag<br>(iv) MF screening<br>2. Drug Distribution<br>3. Adverse Events Survey (active)<br>3. Adverse Events Survey (passive)	2400 HHS	15	6	60							A	A	A	A	A	A	A	A	A	A	A	A	A								
$8	Rapid Response team visits											DHO	DHO	DHO	DHO	DHO	DHO	DHO	DHO	DHO	DHO	DHO	DHO	DHO								
$9	Post-Treatment Survey - Acceptability	400 persons	2	2	30																			B	B							
$10	Post (12 M) -Treatment assessment - Mf and Ag survey	2400 HHS	15	2	19																							C	C	C	C	C
$11	Slide processing and examination	990 Agpos	1	3	20																							C	C	C	C	C
$12	Data analysis and report writing																															

Team composition

Team A : Physician(1no.); Technician C (3nos.: 1 for blood sampling, 1 for FTS reading and 1 for Data entry); Nurse/Field Worker (1no.) Field Assistants (1no.)

Team B : Sociologist (1); Junior Social Worker (1)

Team C: Technician C (2no.); Field Worker (1no.); Field Assistant 1no.); DEO (1no.)

DHO : Distirct Health Officials

10. BUDGET

Budget Heads	Amount in INR	Amount in USD
Personnel	₹ 238,38,293.00	$3,50,929.54
Field allowances	₹ 10,80,000.00	$15,898.95
Equipment	₹ 15,22,500.00	$22,413.11
Supplies	₹ 4,02,000.00	$5,917.94
Contingency	₹ 2,80,400.00	$4,127.84
Mobility	₹ 36,00,000.00	$52,996.51
Communication	₹ 1,26,000.00	$1,854.88
Field lab	₹ 15,84,000.00	$23,318.46
Miscellaneous (Insurance policy)	₹ 2,14,700.00	$3,160.65
Review and steering committee meetings (ICMR)	₹ 12,58,200.00	$18,522.28
	₹ 339,06,093.00	$4,99,140.18

11. REFERENCES

1. World Health Organization (1997). Elimination of lymphatic filariasis as a public health problem - resolution of the executive board of the WHO.

2. National Vector Borne Disease Control Programme (2015). http://nvbdcp.gov.in/filariasis-new.html.

3. World Health Organization (2002). Global Programme to Eliminate Lymphatic Filariasis. Annual Report on Lymphatic Filariasis. Geneva: WHO.

4. Thomsen EK, Sanuku N, Baea M, Satofan S, Maki E, Lombore B, Schmidt MS, Siba PM, Weil GJ, Kazura JW, Fleckenstein LL and King CL (2015). Efficacy, Safety, and Pharmacokinetics of Co-Administered Diethylcarbamazine, Albendazole, and Ivermectin for the Treatment of Bancroftian Filariasis.*Clin Infect Dis. DOI:10.1093/cid/civ882.*

5. Gyapong J, Kumaraswami V, Biswas G, Ottesen EA (2005). Treatment strategies underpinning the global programme to eliminate lymphatic filariasis. *Expert Opin Pharmacother*.: 6 (2), 179-200.

6. Horton J (2000). Albendazole: a review of antihelminthic efficacy and safety in humans. *Parasitology:* 121 (Suppl.), S113-S132.

7. Goa KL, Mctavish D, Clissold SP (1991). Ivermectin: a review of its antifilarial activity, pharmacokinetic properties and clinical efficacy in onchocerciasis. *Drugs:* 42 (4), 640-658.

8. Edwards G (2003). Ivermectin. Does P-glycoprotein play a role in neurotoxicity? *Filaria J:* 2 (Suppl. 1), S8.

9. Ottesen EA, Campbell WC (1994). Ivermectin in human medicine. *J. Antimicrob. Chemother.*: 34 (2), 195-203.

10. Awadzi K, Edwards G, Duke BO *et al.* (2003). The co-administration of ivermectin and albendazole-safety, pharmacokinetics and efficacy against *Onchocerca volvulus*. *Ann. Med. Parasitol.*: 97(2), 165-178.

11. Ottesen EA (1985). Efficacy of diethylcarbamazine in eradicating infection with lymphatic dwelling filariae in humans. *Rev. Infect. Dis.*: 7 (3), 341-356.

12. Nores J, Dreyer G, Santos A, Mendes VG, Medeiros Z, Addiss D (1997). Assessment of the efficacy of diethylcarbamazine on adult *Wuchereria bancrofti* in vivo. *Trans. R. Soc. Trop. Med. Hyg.*: 91 (1), 78-81.

Table 1 Mf prevalence in sentinel and spot check sites in Yadgir district in 2014 and 2015

PHC	Sub-centre	Village	Type	Population	Mf % (2014)	Mf % (2015)
Dornalli	Dornalli	Doranahalli	Sentinel	1369	5.38	3.09
Konkal	Nazarpur	Nazarpur	Sentinel	2131	2.20	1.18
Petammapur	Petammapur	Petammapur	Sentinel	3820	1.90	3.30
GGH Shorapur	Shorapur	Ranganpet	Sentinel	707	1.97	0.40
Yelheri	Yelheri	Yelheri	Spot-check	3575	2.53	ND
Sagar	Sagar	Sagar	Spot-check	6391	2.95	ND
Devargonal	Devargonal	Devargonal	Spot-check	3300	3.00	ND
Yadgir urban	NA	Kalachaputra	Spot-check	NA	0.58	ND
Shahapur	Shahapur	Shahapur town	Spot-check	NA	ND	1.38
Kandakur	NA	Gunjanur	Spot-check	NA	ND	0.79
Yeleri	NA	Kaladelagundi	Spot-check	NA	ND	0.92
Chattanahalli	NA	Khanapur	Spot-check	NA	ND	1.65

NA: Not available
ND: Not done

Table 2 Drug dosage for DA arm (as per national Guidelines)

Age (Years)	DEC		Albendazole	
	Dose (mg)	No. of 100 mg tablets	Dose (mg)	No. of 400 mg tablets
Less than 2	0	0	0	0
2 - 5	100	1	400	1
6 - 14	200	2	400	1
15 +	300	3	400	1

Table 3 Weight based dosage for ivermectin (all persons of age > 5 years with weight ≥ 15 Kg)

Weight (kg)	Ivermectin (mg)	No. tablets (3 mg)	**Rounded No. tablets (3 mg)**
< 15	1.8	0.6	**0**
15	3.0	1.0	
16	3.2	1.1	
17	3.4	1.1	
18	3.6	1.2	
19	3.8	1.3	**1**
20	4.0	1.3	
21	4.2	1.4	
22	4.4	1.5	
23	4.6	1.5	
24	4.8	1.6	
25	5.0	1.7	
26	5.2	1.7	
27	5.4	1.8	
28	5.6	1.9	
29	5.8	1.9	
30	6.0	2.0	
31	6.2	2.1	**2**
32	6.4	2.1	
33	6.6	2.2	
34	6.8	2.3	
35	7.0	2.3	
36	7.2	2.4	
37	7.4	2.5	
38	7.6	2.5	

Weight (kg)	Ivermectin (mg)	No. tablets (3 mg)	**Rounded No. tablets (3 mg)**
39	7.8	2.6	
40	8.0	2.7	
41	8.2	2.7	
42	8.4	2.8	
43	8.6	2.9	
44	8.8	2.9	
45	9.0	3.0	
46	9.2	3.1	**3**
47	9.4	3.1	
48	9.6	3.2	
49	9.8	3.3	
50	10.0	3.3	
51	10.2	3.4	
52	10.4	3.5	
53	10.6	3.5	
54	10.8	3.6	
55	11.0	3.7	
56	11.2	3.7	
57	11.4	3.8	
58	11.6	3.9	
59	11.8	3.9	
60	12.0	4.0	**4**
61	12.2	4.1	
62	12.4	4.1	
63	12.6	4.2	
64	12.8	4.3	
65	13.0	4.3	
66	13.2	4.4	
≥67	13.4	4.5	

Table 4 Drug requirements for the two arms, IDA and DA

Drug	Age group (Years)	Population	No. of tablets per person	Total no. Tablets	Additional tablets *	Grand total (Tablets)
DEC 100 mg	All $\geq$ 2 years	12000	2.5	30000	7500	37500
Albendazole 400 mg	All $\geq$ 2 years	12000	1	12000	3000	15000
Ivermectin **3mg**[#]	All $\geq$5 years	6000	1-4*	21600	5400	27000

[#] Based on 200 µg per Kg body weight

* Required for retreatment of positives and marginal increase in population size during the selection of villages.

Appendix 2 Background of the study drugs

ALB causes degenerative alterations in the tegument and intestinal cells of the worm by binding to the colchicine-sensitive site of tubulin, thus inhibiting its polymerization or assembly into microtubules [6]. The loss of cytoplasmic microtubules leads to impaired uptake of glucose by larval and adult stages of the parasite, and depletes glycogen stores. Degenerative changes in endoplasmic reticulum and mitochondria of the germinal layer, and the subsequent release of lysosomal enzymes result in decreased production of adenosine triphosphate, which is the source of energy required for survival of the helminth. Due to diminished energy production, the parasite is immobilized and eventually dies.

IVM is an avermectin compound of macrocyclic lactones derived from the bacterium *Streptomyces avermitilis* [7]. The mechanism by which IVM kills LF microfilariae is not known with certainty, but the drug interferes with glutamate gated ion channels that can affect parasite contractility and release of immunomodulatory molecules by the parasite [7]. IVM also has a direct effect on the central nervous system and muscle function as it enhances strength of inhibitory neurotransmission pathways. The main concern with the use of IVM in animals and humans is neurotoxicity, which can be manifest as ataxia. Neurotoxicity is usually associated with prolong therapy with the drug and has not been observed in humans given single dose IVM for LF or other parasitic infections [8]. IVM has been used to treat millions of people with LF and onchocerciasis [8]. Peak IVM serum concentrations are reached approximately 4-5 hours after administration. The half-life of IVM in various populations ranges from 12 to 56 hours [9]. There is no evidence of drug: drug interaction between ALB and IVM [10]. IVM can cause nausea, dizziness and occasionally pruritus, but these are infrequent, transient and usually mild. Major side effects occur with heavy infections of *L. loa*; however, this parasite is not endemic in India.

DEC is an anthelminthic drug that is structurally distinct from ALB and IVM [11]. DEC inhibits arachidonic acid metabolism by LF, and inducible nitric oxide synthase and the cyclooxygenase pathway may be essential for activity *in vivo* [11]. DEC also has anti-inflammatory properties. The mechanisms of active of DEC remain poorly understood. Its ability to kill Mf and adult worm depends on the host immune responses since the drug has little direct activity on parasites *in vitro*. The drug has potent activity against LF microfilaria. DEC has about 50-70% efficacy in killing or sterilization of adult worms [12]. The drug is rapidly absorbed from the gastrointestinal tract, has a serum half-life of 12 to 14 hours, and is excreted in the urine with little modification by liver metabolism. There are few side effects to drug other than killing of adult worms.

Appendix 3 Study flow in each arm

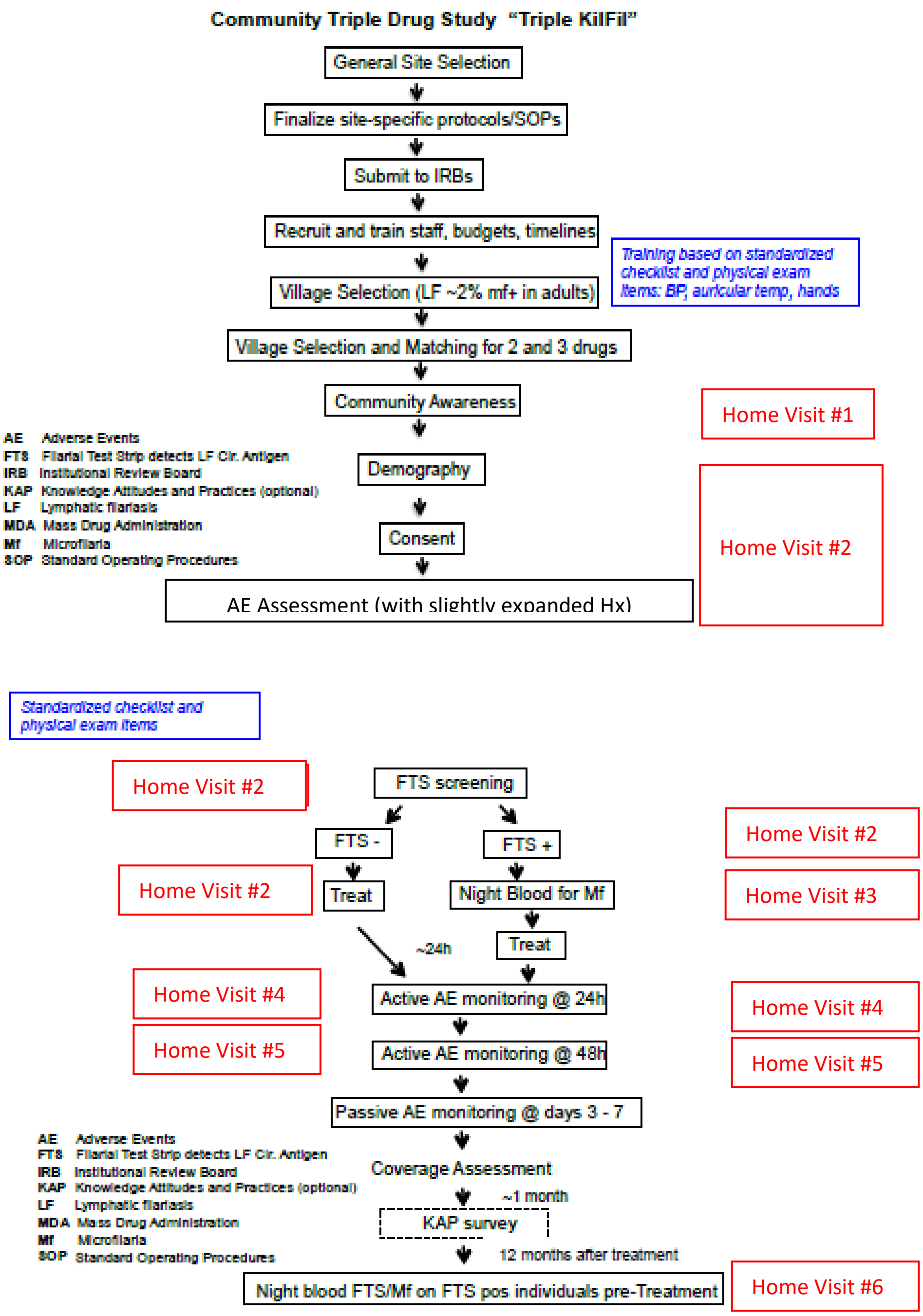

[Total 'n' in both arms together=12000 (2 x 6000)]

Appendix 4. INFORMATION SHEET AND CONSENT FORM

TRIPLE DRUG THERAPY FOR LYMPHATIC FILARIASIS STUDY

Person ID (Barcode):

Project title: A community based study, to compare the safety, efficacy and acceptability of a triple drug regimen (Ivermectin, Diethylcarbamazine and Albendazole) with a two-drug regimen (Diethylcarbamazine and Albendazole) for lymphatic filariasis elimination programme

Part A - Information sheet

Your area is endemic for lymphatic filariasis. A National programme to eliminate this disease is implemented from 2004. Anti-filarial tablets (DEC + Albendazole) are distributed to all the people above 2 years of age in your area once a year. So far the tablets were distributed twelve times, but the infection levels have not reduced to the desired level i.e one in 100 individuals in your area. It is time to find out alternate methods of improving the efficacy of the treatment. A trial conducted elsewhere with tablets of ivermectin, DEC and albendazole showed that this regimen is highly useful to clear the infection and achieve total interruption of spread. World Health Organisation and Directorate of National Vector Borne Diseases are awaiting for the results of a community trial with this new drug regimen so that this strategy can be introduced in the programme to clear the persistent infection in your district and the districts like yours. Initially you and your child will be tested for filarial infection using antigen test (using a few drops (75µl) of finger prick blood sample). If found positive, you and your child will be screened for Mf in the night of the same day (again using finger prick blood sample of a few drops (60µl)). All the individuals $\geq$ 5 years age in your village will be tested for filarial infection using the same method and subsequently administered with either three drug regimen or two drug regimen based on the measured body weight under the supervision of medical personnel. All those individuals administered drugs will be closely monitored for side effects at 24, 48 hours and up to 7 days. They will be provided treatment (if necessary) to manage the side effects. You and your child will be re-tested after one year for filarial infection. Appropriate treatment will be provided if you or your child continue to show positive.

Voluntary participation:

The following paragraphs will provide you the details of the methods of study and you are free to clarify any information on these procedures. It is entirely your option for your or your child's participation in this study or not.

Procedures:

A few drops (75 µl) of blood will be drawn by finger prick method and tested for filarial infection using antigen test. Disposable lancet and disinfectants will be used. The result will be shown within 10

Person ID (Barcode):

minutes. If the test shows positive for filarial antigen, you will be tested for mf in the night of the same day, using a finger prick night blood sample. 60 µl blood sample will be drawn for this purpose. You will be asked questions related to your health conditions including those related to filariasis and examined by a physician, if necessary. You will be given antifilarial tablets, either DEC and Albendazole or DEC, Albendazole and Ivermectin. The number of tablets given will depend on your body weight which will be measured using a weighing machine. You will be asked to swallow the tablets with a glass of water in front of the health staff after ensuring that you have taken food before taking tablets.

Potential risks:

This study does not have any foreseeable risks. As disposable lancets with disinfectants will be used there is no potential risk. No blood samples will be stored for further test. No serious adverse effect following treatment is foreseen. Some people experience side effects after treatment for filariasis such as fever, headache and fatigue, and these are mainly related to the death of the worms. Most side effects are mild and last only 1 or 2 days which will disappear spontaneous with or without treatment. All treated individuals will be closely followed by the Medical team for the first two days following treatment and side effects will be managed effectively. The team will be in the village up to 7 days to whom you can approach for any discomfort. You will be referred for treatment at free of cost in the hospital if necessary. In the rare event of developing some side effects due to drug administration leading to loss of wages, you will be compensated based on the physician report. In case of severe side effects, you will be compensated as per the Central Drug Standard Control Organization (CDSCO) guidelines.

Benefits to taking part in the study:

Participants, who sign an informed consent, will be treated with the anti-filarial drugs. LF transmission to the community will be reduced by participation in either treatment arm. A broader community benefit may be facilitated by the triple drug regimen as it is believed the triple drug regimen has the potential to markedly reduce the number of Mass Drug Administrations needed to achieve transmission interruption and elimination of LF. Both regimens provide treatment for intestinal worms, and the triple drug treatment has the added benefit of providing an effective treatment for scabies. If the triple drug intervention proves successful, the triple therapy is likely to be adopted in India and many LF endemic areas globally.

Appendix 4. INFORMATION SHEET AND CONSENT FORM
TRIPLE DRUG THERAPY FOR LYMPHATIC FILARIASIS STUDY

Person ID (Barcode):

Participation, Cost and right to withdraw

Participation is voluntary and you may decline participation without consequences. There will be no cost to participate in the study and you will not be paid for your participation. The study will cover cost associated with laboratory test, study drugs, and clinical monitoring. You have the right to withdraw yourself from the study, in case you decide to stop participating,

Confidentiality of records:

Your identity and information provided by you will be kept confidential.

Contacts for questions or problems:

You also have the liberty to access the investigators for further clarifications, doubts about the study. You can fully satisfy yourself regarding the information about the study before consenting. You are free to contact the following officials for any details and clarifications.

1. Dr. P. Jambulingam 9443234551
2. Dr. R. J. De Britto 9444419219
3. Dr. S. Subramanian 9487062220
4. Mrs. A. Srividya 9894404690
5. Dr. B. Nandha 9442550438
6. Dr. K. Krishnamoorthy 9442787436

Appendix 4. INFORMATION SHEET AND CONSENT FORM
TRIPLE DRUG THERAPY FOR LYMPHATIC FILARIASIS STUDY

Person ID (Barcode):

Project title: A community based study, to compare the safety, efficacy and acceptability of a triple drug regimen (Ivermectin, Diethylcarbamazine and Albendazole) with a two-drug regimen (Diethylcarbamazine and Albendazole) for lymphatic filariasis elimination programme

Part B (adults/ adolescents)

Written Consent form for participating in the study

I have read the information given in the information sheet, and fully understood the details therein. I have also been explained by the Principal investigator or his duly authorized representative the full details, and having fully satisfied myself with the explanation given, of my own volition with full sense / awareness, give my consent for self for all screening procedures that are required for screening for infection due to filariasis. Also I give my consent for treating with the drugs for clearing infection.

I am also fully aware of my right to withdraw myself from the study, and in case should I stop participating, that I will be given treatment for the disease or will be referred for appropriate treatment.

Given this day…………..in the month of ……………in the year………

Signed in my presence Signature of the individual

Signature of the witness

1

Address Address

……………………………………………………… ……………………………………………………………….

……………………………………………………… ……………………………………………………………….

……………………………………………………… ……………………………………………………………….

2

Address

……………………………………………………………………………………….

……………………………………………………………………………………….

Name of the informant Signature of the informant

Appendix 4. INFORMATION SHEET AND CONSENT FORM
TRIPLE DRUG THERAPY FOR LYMPHATIC FILARIASIS STUDY

Person ID (Barcode):

Part C – Assent Form for children in the age class 7-17 years

Project title: A community based study, to compare the safety, efficacy and acceptability of a triple drug regimen (Ivermectin, Diethylcarbamazine and Albendazole) with a two-drug regimen (Diethylcarbamazine and Albendazole) for lymphatic filariasis elimination programme

Investigator: Dr.P.Jambulingam

We are doing a research study to identify more effective drug regimen to treat and prevent lymphatic filariasis in your area. All the people who are eligible for drug administration will be involved in this study. If you decide that you want to be part of this study, we will tell you what other kinds of treatments there are for you.

There are some things about this study you should know and the relevant information are given in the Part A of this document. By participating in this study you will benefit directly by getting cleared of infection if you are infected or indirectly in preventing new infections.

When we are finished with this study we will write a report about what was learned and this report will not include your name or that you were in the study.

Your participation is voluntary. You can opt for withdrawing your participation at any point of the study. Your parents know about the study too and consent for your participation will be obtained from them.

If you decide you want to be in this study, please sign your name.

I, ___________________________________, want to be in this research study.

_____________________________________ ______

(Sign your name here) (Date)

Appendix 4. INFORMATION SHEET AND CONSENT FORM
TRIPLE DRUG THERAPY FOR LYMPHATIC FILARIASIS STUDY

Person ID (Barcode):

Part D - Parental Informed Consent form

Your child has been invited to join the proposed research study explained in part A. The decision to let your child join, or not to join, is purely voluntary.

In this research study, we are investigating the efficacy and safety of a new drug regimen to eliminate lymphatic filariasis.

Written consent form for participation of children in the study

I have read the information given in the information sheet, and fully understood the details therein. I have also been explained by the Principal investigator / duly authorized representative the full details, and having fully satisfied myself with the explanation given, of my own volition with full sense / awareness, give my consent for my son / my daughter for all screening procedures that are required for screening for infection due to filariasis. Also I give my consent for treating my child with the drugs for clearing infection.

I am also fully aware of my right to withdraw my ward from the study, and in case should I stop my child participating, that my ward will be given treatment for the disease or will be referred for appropriate treatment.

Permission for a Child to Participate in Research

As parent or legal guardian, I authorize ________________________________ (child's name) to become a participant in the research study described in this form.
Child's Date of Birth: ______________________

Parent or Legal Guardian's Signature Date

Signed in my presence of:

Signature of the witness: 1 Signature of the Witness: 2

Address Address

……………………………………………………. ………………………………………………………….
……………………………………………………. ………………………………………………………….
……………………………………………………. ………………………………………………………….

Name of the informant Signature of the informant

Appendix 4. INFORMATION SHEET AND CONSENT FORM
TRIPLE DRUG THERAPY FOR LYMPHATIC FILARIASIS STUDY

Person ID (Barcode):

ಪ್ರಾಜೆಕ್ಟ್ ಶೀರ್ಷಿಕೆ: ಒಂದು ಸಮುದಾಯ ಆಧಾರಿತ ಅಧ್ಯಯನವು ಎರಡು ಔಷಧ ಕಟ್ಟುಪಾಡು (ಡಿಎಥ್ಯ್ಲ್ಚರ್ಬಮಶಿನೆ ಮತ್ತು ಅಲ್ಬೆನ್ದಶೊಲೆ) ದುಗ್ದನಾಳ ಫಿಲಾರಿಯಾಸಿಸ್ ಎಲಿಮಿನೇಷನ್ ಕಾರ್ಯಕ್ರಮದಲ್ಲಿ ಜೊತೆ ಸುರಕ್ಷತೆ, ದಕ್ಷತೆಯ ಮತ್ತು ಒಂದು ಟ್ರಿಪಲ್ ಔಷಧ ಕಟ್ಟುಪಾಡು (ಇವೆರ್ಮೆಕ್ಟಿನ್,ಡಿಎಥ್ಯ್ಲ್ಚರ್ಬಮಶಿನೆ ಮತ್ತು ಅಲ್ಬೆನ್ದಶೊಲೆ) ಸ್ವೀಕಾರವನ್ನು ಹೋಲಿಸಿ

ಭಾಗ ಒಂದು - ಮಾಹಿತಿ ಹಾಳೆ

ನಿಮ್ಮ ಪ್ರದೇಶದಲ್ಲಿ ದುಗ್ದನಾಳ ಫಿಲಾರಿಯಾಸಿಸ್ ಫಾರ್ ಸ್ಥಳೀಯ ಹೊಂದಿದೆ. ಈ ಕಾಯಿಲೆಯ ನಿರ್ಮೂಲನೆಗೆ ರಾಷ್ಟ್ರೀಯ ಕಾರ್ಯಕ್ರಮ 2004 ವಿರೋಧಿ ಫಿಲೇರಿಯಾದ ಔಷಧಗಳು (ಡಿಸೆಂಬರ್ + ಅಲ್ಬೆನ್ದಶೊಲೆ) ಎಲ್ಲಾ ಜನರು (ಮಾಸ್ ಡ್ರಗ್ ಅಡ್ಮಿನಿಸ್ಟ್ರೇಷನ್-MDA), ವರ್ಷಕ್ಕೊಮ್ಮೆ ಹಂಚಲಾಗುತ್ತದೆ ರಿಂದ ಅಳವಡಿಸಲಾಗಿದೆ. ಇಲ್ಲಿಯವರೆಗೆ ಹನ್ನೆರಡು ಸುತ್ತುಗಳ ಮುಗಿದಿಲ್ಲ ಆದರೆ ಸೋಂಕು ಮಟ್ಟಗಳು 1% ಕ್ಕಿಂತಲೂ ಕಡಿಮೆ ಗುರಿ ತಲುಪಿತು ಮಾಡಿಲ್ಲ. ಇದು ವ್ಯಾಪ್ತಿ ಹೆಚ್ಚಿಸುವ ಜೊತೆಗೆ ಔಷಧ ಪರಿಣಾಮಕಾರಿತ್ವದ ಸುಧಾರಿಸುವ ವಿಧಾನಗಳನ್ನು ಗುರುತಿಸಲು ಸಮಯ. ಇವೆರ್ಮೆಕ್ಟಿನ್ ಏಕೈಕ ಡೋಸ್ ನಡೆಸಿದ ಪ್ರಯೋಗ, ಡಿಸೆಂಬರ್ ಮತ್ತು ಅಲ್ಬೆನ್ದಶೊಲೆ ಈ ಕಟ್ಟುಪಾಡು ಮಿಕ್ರೊಫಿಳರೆಮಿಕ್ ತೆರವುಗೊಳಿಸಲು ಮತ್ತು ಪ್ರಸರಣ ಒಟ್ಟು ತಡೆ ಸಾಧಿಸಲು ಹೆಚ್ಚು ಉಪಯುಕ್ತ ತೋರಿಸಿದರು. ನಿಮ್ಮ ಜಿಲ್ಲೆಯ ನಿರಂತರ ಸೋಂಕು ಮತ್ತು ನಿಮ್ಮ ಹಾಗೆ ಜಿಲ್ಲೆಗಳಲ್ಲಿ ತೆರವುಗೊಳಿಸಲು ಈ ತಂತ್ರ ಬಳಸಲು ಆದ್ದರಿಂದ ವಿಶ್ವ ಆರೋಗ್ಯ ಸಂಸ್ಥೆ ಮತ್ತು ರಾಷ್ಟ್ರೀಯ ವೆಕ್ಟರ್ ಬೋರ್ನ್ ಡಿಸೀಸಸ್ ನಿರ್ದೇಶನಾಲಯ ಫಲಿತಾಂಶಗಳು ಸಮುದಾಯ ವಿಚಾರಣೆಯ ಈ ಹೊಸ ಔಷಧ ಕಟ್ಟುಪಾಡು ಫಾರ್ ಕಾಯುತ್ತಿವೆ. ಆರಂಭದಲ್ಲಿ ನೀವು ಮತ್ತು ನಿಮ್ಮ ಮಗು ಪ್ರತಿಜನಕ ತಪಾಸಣೆ (ಕೆಲವು ಹನಿಗಳನ್ನು (ಫಿಂಗರ್ ಚುಚ್ಚು ರಕ್ತದ ನಮೂನೆಯ 75µl) ಬಳಸಿ) ಬಳಸಿಕೊಂಡು ಫಿಲೇರಿಯಾದ ಸೋಂಕು ಪರೀಕ್ಷೆ ನಡೆಯಲಿದೆ. ಧನಾತ್ಮಕ ಕಂಡು ವೇಳೆ, ನೀವು ಮತ್ತು ನಿಮ್ಮ ಮಗು ಅದೇ ದಿನದ ರಾತ್ರಿ ಎಂಎಫ್ ಪ್ರದರ್ಶಿಸಲಾಯಿತು ನಡೆಯಲಿದೆ (ಮತ್ತೆ ಕೆಲವು ಹನಿಗಳನ್ನು ಬೆರಳನ್ನು ಚುಚ್ಚಿಕೊಂಡು ರಕ್ತದ ಮಾದರಿಯನ್ನು (60µl) ಬಳಸಿ). ಎಲ್ಲಾ ವ್ಯಕ್ತಿಗಳು ನಿಮ್ಮ ಹಳ್ಳಿಯಲ್ಲಿ > 5 ವರ್ಷ ಅದೇ ವಿಧಾನವನ್ನು ಬಳಸಿಕೊಂಡು ಫಿಲೇರಿಯಾದ ಸೋಂಕು ಪರೀಕ್ಷೆ ನಡೆಯಲಿದೆ ಮತ್ತು ತರುವಾಯ ಮೂರು ಔಷಧ ಕಟ್ಟುಪಾಡು ಅಥವಾ ವೈದ್ಯಕೀಯ ಸಿಬ್ಬಂದಿ ಮೇಲ್ವಿಚಾರಣೆಯಲ್ಲಿ ಹೆಸರು ದೇಹ ತೂಕದ ಆಧಾರದ ಮೇಲೆ ಎರಡು ಔಷಧ ಕಟ್ಟುಪಾಡು ಎರಡೂ ಆಡಳಿತ. ಎಲ್ಲಾ ಆಡಳಿತ ಔಷಧಗಳ ಆ ವ್ಯಕ್ತಿಗಳು ನಿಕಟವಾಗಿ 7 ದಿನಗಳ 24, 48 ಗಂಟೆಗಳ ಅಡ್ಡಪರಿಣಾಮಗಳು ಮತ್ತು ಮೇಲ್ವಿಚಾರಣೆ ಮಾಡಲಾಗುತ್ತದೆ. ಅವರು ಅಡ್ಡ ಪರಿಣಾಮಗಳನ್ನು ನಿರ್ವಹಿಸಲು (ಅಗತ್ಯವಿದ್ದರೆ) ಒದಗಿಸಿದ ಚಿಕಿತ್ಸೆ ಮಾಡಲಾಗುತ್ತದೆ. ನೀವು ಮತ್ತು ನಿಮ್ಮ ಮಗು ನಡೆಯಲಿದೆ ಫಿಲೇರಿಯಾದ ಸೋಂಕು ಒಂದು ವರ್ಷದ ನಂತರ ಮರು ಪರೀಕ್ಷೆ. ನೀವು ಅಥವಾ ನಿಮ್ಮ ಮಗುವಿಗೆ ಧನಾತ್ಮಕ ತೋರಿಸಲು ಮುಂದುವರಿದರೆ ಸೂಕ್ತ ಚಿಕಿತ್ಸೆ ಒದಗಿಸಲಾಗುವುದು.

ವಾಲಂಟರಿ ಭಾಗವಹಿಸುವಿಕೆ:

ಕೆಳಗಿನ ಪ್ಯಾರಾಗಳು ನೀವು ಅಧ್ಯಯನ ವಿಧಾನಗಳ ವಿವರಗಳನ್ನು ಮತ್ತು ನೀವು ಈ ಕಾರ್ಯವಿಧಾನಗಳು ಯಾವುದೇ ಮಾಹಿತಿ ಸ್ಪಷ್ಟನೆ ಉಚಿತ. ಇದು ಸಂಪೂರ್ಣವಾಗಿ ಈ ಅಧ್ಯಯನ ಅಥವಾ ನಿಮ್ಮ ಅಥವಾ ನಿಮ್ಮ ಮಗುವಿನ ಭಾಗವಹಿಸುವಿಕೆ ನಿಮ್ಮ ಆಯ್ಕೆಯಾಗಿದೆ.

ವಿಧಾನಗಳು:

Person ID (Barcode):

ಕೆಲವು ಹನಿಗಳನ್ನು (75 µl) ರಕ್ತದ ಬೆರಳು ಚುಚ್ಚು ವಿಧಾನ ಎಳೆಯುವ ಮತ್ತು ಪ್ರತಿಜನಕದ ಪರೀಕ್ಷೆಯನ್ನು ಬಳಸಿಕೊಂಡು ಫಿಲೇರಿಯಾದ ಸೋಂಕು ಪರೀಕ್ಷೆ ನಡೆಯಲಿದೆ. ಡಿಸ್ಪೋಸಬಲ್ ಲ್ಯಾನ್ಸೆಟ್ ಮತ್ತು ಸೋಂಕು ಬಳಸಲಾಗುತ್ತದೆ. ಪರಿಣಾಮವಾಗಿ 10 ನಿಮಿಷಗಳಲ್ಲಿ ತೋರಿಸಲಾಗುತ್ತದೆ. ಟೆಸ್ಟ್ ಫಿಲೇರಿಯಾದ ಪ್ರತಿಜನಕ ಧನಾತ್ಮಕ ತೋರಿಸುತ್ತದೆ, ನೀವು ಎಂಎಫ್ ಅದೇ ದಿನ ರಾತ್ರಿ, ಒಂದು ಬೆರಳು ಚುಚ್ಚು ರಾತ್ರಿ ರಕ್ತದ ಮಾದರಿಯನ್ನು ಬಳಸಿಕೊಂಡು ಪರೀಕ್ಷೆ ನಡೆಯಲಿದೆ. 60 µl ರಕ್ತದ ಮಾದರಿಯನ್ನು ಈ ಉದ್ದೇಶಕ್ಕಾಗಿ ಡ್ರಾ ನಡೆಯಲಿದೆ. ನೀವು ಫಿಲಾರಿಯಾಸಿಸ್ ಸಂಬಂಧಿಸಿದ ಮತ್ತು ಅಗತ್ಯವಿದ್ದರೆ, ವೈದ್ಯ ಪರೀಕ್ಷಿಸಿ ಸೇರಿದಂತೆ ನಿಮ್ಮ ಆರೋಗ್ಯ ಸಮಸ್ಯೆಗಳನ್ನು ಸಂಬಂಧಿಸಿದ ಪ್ರಶ್ನೆಗಳನ್ನು ಕೇಳಲಾಗುತ್ತದೆ. ನೀವು ಅಂತಿ-ಫೈಲರಿಅಲ್ ಮಾತ್ರೆಗಳು ನೀಡಲಾಗುವುದು, ಡಿಸೆಂಬರ್ ಎರಡೂ ಮತ್ತು ಅಲ್ಬೆನ್ದಶೊಲೆ ಅಥವಾ ಡಿಸೆಂಬರ್, ಅಲ್ಬೆನ್ದಶೊಲೆ ಮತ್ತು ಇವೆರ್ಮೆಕ್ಟಿನ್ ಕಾಣಿಸುತ್ತದೆ. ಸಂಖ್ಯೆ ಕೊಟ್ಟಿರುವ ಒಂದು ತೂಕ ಯಂತ್ರ ಬಳಸಿಕೊಂಡು ಅಳೆಯಲಾಗುವುದು ನಿಮ್ಮ ದೇಹದ ತೂಕ ಅವಲಂಬಿಸಿರುತ್ತದೆ ಮಾತ್ರೆಗಳು. ನೀವು ಮಾತ್ರೆಗಳು ತೆಗೆದುಕೊಳ್ಳುವ ಮೊದಲು ಆಹಾರ ತೆಗೆದುಕೊಂಡ ಖಾತರಿ ನಂತರ ಆರೋಗ್ಯ ಸಿಬ್ಬಂದಿ ಮುಂದೆ ನೀರಿನ ಗಾಜಿನ ಮಾತ್ರೆಗಳು ನುಂಗಲು ಕೇಳಲಾಗುತ್ತದೆ.

ಸಂಭಾವ್ಯ ಅಪಾಯಗಳನ್ನು:

ಈ ಅಧ್ಯಯನವು ಯಾವುದೇ ನಿರೀಕ್ಷಿತ ಅಪಾಯಗಳನ್ನು ಹೊಂದಿಲ್ಲ. ಸೋಂಕು ಬಳಸಬಹುದಾದ lancets ಬಳಸಲಾಗುತ್ತದೆ ಯಾವುದೇ ಸಂಭಾವ್ಯ ಅಪಾಯವಿದೆ. ಯಾವುದೇ ರಕ್ತ ಮಾದರಿಗಳನ್ನು ಮತ್ತಷ್ಟು ಪರೀಕ್ಷೆಗೆ ಸಂಗ್ರಹಿಸಲಾಗುವುದು. ಚಿಕಿತ್ಸೆ ನಂತರ ಯಾವುದೇ ಗಂಭೀರ ಪ್ರತಿಕೂಲ ಪರಿಣಾಮ ಮುಂಚೆಯೇ ಇದೆ. ಕೆಲವರು ಜ್ವರ, ತಲೆನೋವು ಮತ್ತು ನಿಶ್ಯಕ್ತಿಯಂತಹ ಫಿಲಾರಿಯಾಸಿಸ್ ಚಿಕಿತ್ಸೆ ನಂತರ ಅಡ್ಡಪರಿಣಾಮಗಳು ಅನುಭವಕ್ಕೆ, ಮತ್ತು ಈ ಹುಳುಗಳು ಸಾವಿಗೆ ಸಂಬಂಧಿತವಾಗಿರುವ ಕಾರಣದಿಂದ. ಅತ್ಯಂತ ಅಡ್ಡಪರಿಣಾಮಗಳು ಸೌಮ್ಯ ಮತ್ತು ಕೇವಲ 1 ಅಥವಾ 2 ದಿನಗಳ ಕಾಲ. ಎಲ್ಲಾ ಚಿಕಿತ್ಸೆ ವ್ಯಕ್ತಿಗಳು ಹಿಂದೆಯೇ ಮತ್ತು ಅಡ್ಡ ಪರಿಣಾಮಗಳು ಪರಿಣಾಮಕಾರಿಯಾಗಿ ನಿರ್ವಹಿಸುತ್ತಿದ್ದ ನಡೆಯಲಿದೆ. ಕಾರಣ ವೇತನ ನಷ್ಟ ಪ್ರಮುಖ ಔಷಧ ಆಡಳಿತ ಕೆಲವು ಅಡ್ಡ ಪರಿಣಾಮಗಳು ಅಭಿವೃದ್ಧಿ ಅಪರೂಪದ ಸಂದರ್ಭದಲ್ಲಿ, ನೀವು ಪರಿಹಾರ ಆಗುತ್ತದೆ. ಇದಲ್ಲದೆ, ಅಡ್ಡ ಪರಿಣಾಮಗಳ ನಿರ್ವಹಣೆ ಕಡೆಗೆ ಎಲ್ಲಾ ನಿಮ್ಮ ಚಿಕಿತ್ಸೆ ವೆಚ್ಚ ಅಧ್ಯಯನ ಸುರಕ್ಷಿತವಾಗಿರುತ್ತಾನೆ. ತೀವ್ರ ಅಡ್ಡ ಪರಿಣಾಮಗಳ ಸಂದರ್ಭದಲ್ಲಿ, ನೀವು ಸೆಂಟ್ರಲ್ ಡ್ರಗ್ ಪ್ರಮಾಣಿತ ನಿಯಂತ್ರಣ ಸಂಸ್ಥೆ (CDSCO) ಮಾರ್ಗಸೂಚಿಗಳ ಪ್ರಕಾರ ಪರಿಹಾರ ಆಗುತ್ತದೆ.

ಅಧ್ಯಯನದಲ್ಲಿ ಭಾಗವಹಿಸಿದ ಪ್ರಯೋಜನಗಳು:

ಒಂದು ಸಮ್ಮತಿ ಸಹಿ ಭಾಗವಹಿಸಿದವರು, ವಿರೋಧಿ ಫಿಲೇರಿಯಾದ ಮಾದಕ ಪರಿಗಣಿಸಲಾಗುತ್ತದೆ. ಸಮುದಾಯಕ್ಕೆ ಲ್ಯಾಂಫಾತಿಕ್ ಫಿಲೇರಿಯಾಸಿಸ್ ಪ್ರಸರಣ ಎರಡೂ ಚಿಕಿತ್ಸೆ ತೋಳಿನಲ್ಲಿ ಭಾಗವಹಿಸುವುದರಿಂದ ಕಡಿಮೆಯಾಗುತ್ತದೆ. ಇದು ಟ್ರಿಪಲ್ ಔಷಧ ಕಟ್ಟುಪಾಡು ಪ್ರಸರಣ ತಡೆ ಮತ್ತು ಲ್ಯಾಂಫಾತಿಕ್ ಫಿಲೇರಿಯಾಸಿಸ್ ಎಲಿಮಿನೇಷನ್ ಸಾಧಿಸಲು ಗಮನಾರ್ಹವಾಗಿ ಸಾಮೂಹಿಕ ಔಷಧ ಕೊಡುವಿಕೆ ಸಂಖ್ಯೆ ಕಡಿಮೆಗೊಳಿಸುವ ಸಾಮರ್ಥ್ಯವನ್ನು ಹೊಂದಿದೆ ನಂಬಲಾಗಿದೆ ಎಂದು ಒಂದು ದೊಡ್ಡ ಸಮುದಾಯಕ್ಕೆ ಲಾಭ ಟ್ರಿಪಲ್ ಔಷಧ ಕಟ್ಟುಪಾಡು ಅನುವುಮಾಡಿಕೊಡುತ್ತವೆ ಮಾಡಬಹುದು. ಎರಡೂ ಕಟ್ಟುಪಾಡುಗಳು ಕರುಳಿನ ಹುಳುಗಳು ಚಿಕಿತ್ಸೆ ನೀಡಲು, ಮತ್ತು ಟ್ರಿಪಲ್ ಔಷಧ ಚಿಕಿತ್ಸೆ ತುರಿಗಜ್ಜಿ ಪರಿಣಾಮಕಾರಿ ಚಿಕಿತ್ಸೆ ಒದಗಿಸುವ ಅಧಿಕ ಲಾಭ ಹೊಂದಿದೆ. ಟ್ರಿಪಲ್ ಔಷಧ

Appendix 4. INFORMATION SHEET AND CONSENT FORM
TRIPLE DRUG THERAPY FOR LYMPHATIC FILARIASIS STUDY

Person ID (Barcode):

ಹಸ್ತಕ್ಷೇಪ ಯಶಸ್ವಿ ಸಾಧಿಸುತ್ತಾನೆ ವೇಳೆ, ಟ್ರಿಪಲ್ ಚಿಕಿತ್ಸೆ ಜಾಗತಿಕವಾಗಿ ಭಾರತ ಮತ್ತು ಅನೇಕ ಲ್ಯಾಮ್ಫ್ಯಾತಿಕ್ ಫಿಲೇರಿಯಾಸಿಸ್ ಸ್ಥಳೀಯ ಪ್ರದೇಶಗಳಲ್ಲಿ ದತ್ತು ಸಾಧ್ಯತೆಯಿದೆ.

ವಿಷಯ ಭಾಗವಹಿಸುವಿಕೆ ಮತ್ತು ಕಾಸ್ಟ್

ಭಾಗವಹಿಸುವಿಕೆ ವೈಯಕ್ತಿಕವಾಗಿದ್ದು ಮತ್ತು ನೀವು ಪರಿಣಾಮಗಳನ್ನು ಇಲ್ಲದೆ ಭಾಗವಹಿಸುವಿಕೆ ಕಡಿಮೆಯಾಗಬಹುದು. ಅಧ್ಯಯನದಲ್ಲಿ ಭಾಗವಹಿಸಲು ಯಾವುದೇ ಶುಲ್ಕವಿಲ್ಲ ಮತ್ತು ನೀವು ನಿಮ್ಮ ಭಾಗವಹಿಸುವಿಕೆ ಪಾವತಿ ಮಾಡುವುದಿಲ್ಲ. ಅಧ್ಯಯನ ಪ್ರಯೋಗಾಲಯದ ಪರೀಕ್ಷಾ, ಅಧ್ಯಯನ ಔಷಧಗಳು, ಮತ್ತು ವೈದ್ಯಕೀಯ ಮೇಲ್ವಿಚಾರಣೆ ಸಹವರ್ತಿ ವೆಚ್ಚದ ಕ್ರಮಿಸುತ್ತದೆ. ನೀವು ಅಧ್ಯಯನದ ನಿಮ್ಮನ್ನು ಹಿಂದಕ್ಕೆ ಹಕ್ಕಿದೆ, ಸಂದರ್ಭದಲ್ಲಿ ನೀವು ಭಾಗವಹಿಸುವ ನಿಲ್ಲಿಸಲು ನಿರ್ಧರಿಸಬಹುದು,

ದಾಖಲೆಗಳ ಗೋಪ್ಯತೆಗೆ:

ನಿಮ್ಮ ಗುರುತನ್ನು ಮತ್ತು ನೀವು ಒದಗಿಸಿದ ಮಾಹಿತಿಯನ್ನು ಗೌಪ್ಯವಾಗಿ ಇಡಲಾಗುತ್ತದೆ.

ಪ್ರಶ್ನೆಗಳನ್ನು ಅಥವಾ ಸಮಸ್ಯೆಗಳಿಗೆ ಸಂಪರ್ಕಗಳು:

ನೀವು ಸ್ಪಷ್ಟೀಕರಣಕ್ಕೆ, ಅಧ್ಯಯನ ಸಂಶಯವನ್ನು ಶೋಧಕರು ಪ್ರವೇಶಿಸಲು ಸ್ವಾತಂತ್ರ್ಯ ಹೊಂದಿವೆ. ನೀವು ಸಂಪೂರ್ಣವಾಗಿ ಒಪ್ಪಿಗೆ ಮುನ್ನ ಅಧ್ಯಯನದ ಬಗ್ಗೆ ಮಾಹಿತಿ ಬಗ್ಗೆ ನೀವೇ ಪದಗಳನ್ನು ಮಾಡಬಹುದು. ನೀವು ಯಾವುದೇ ವಿವರಗಳು ಮತ್ತು ಸ್ಪಷ್ಟೀಕರಣ ಕೆಳಗಿನ ಅಧಿಕಾರಿಗಳು ಸಂಪರ್ಕಿಸಲು ಉಚಿತ.

1. ಡಾ ಪಿ ಜಮ್ಬುಲಿನ್ಗಂ 9443234551
2. ಡಾ ಆರ್ ಜೆ ಡಿ ಬ್ರಿತ್ತೋ 9444419219
3. ಡಾ ಎಸ್ ಸುಬ್ರಮಣಿಯನ್ 9487062220
4. ಶ್ರೀಮತಿ ಎ ಶ್ರೀವಿದ್ಯಾ 9894404690
5. ಡಾ ಬಿ ನಂದಾ 9443550438
6. ಡಾ ಕೆ ಕೃಷ್ಣಮೂರ್ತಿ 9442787436

Appendix 4. INFORMATION SHEET AND CONSENT FORM
TRIPLE DRUG THERAPY FOR LYMPHATIC FILARIASIS STUDY

Person ID (Barcode):

ಪ್ರಾಜೆಕ್ಟ್ ಶೀರ್ಷಿಕೆ: ಒಂದು ಸಮುದಾಯ ಆಧಾರಿತ ಅಧ್ಯಯನವು ಎರಡು ಔಷಧ ಕಟ್ಟುಪಾಡು (ಡಿಎಥ್ಯ್ಲ್ಚರ್ಬಮಶಿನೆ ಮತ್ತು ಅಲ್ಬೆನ್ದಶೊಲೆ) ದುಗ್ದನಾಳ ಫಿಲಾರಿಯಾಸಿಸ್ ಎಲಿಮಿನೇಷನ್ ಕಾರ್ಯಕ್ರಮದಲ್ಲಿ ಜೊತೆ ಸುರಕ್ಷತೆ, ದಕ್ಷತೆಯ ಮತ್ತು ಒಂದು ಟ್ರಿಪಲ್ ಔಷಧ ಕಟ್ಟುಪಾಡು (ಇವರ್ಮೆಕ್ಟಿನ್, ಡಿಎಥ್ಯ್ಲ್ಚರ್ಬಮಶಿನೆ ಮತ್ತು ಅಲ್ಬೆನ್ದಶೊಲೆ) ಸ್ವೀಕಾರವನ್ನು ಹೋಲಿಸಿ

ಭಾಗ ಬಿ (ವಯಸ್ಕರಿಗೆ / ಹದಿಹರೆಯದವರಿಗೆ)

ಅಧ್ಯಯನದಲ್ಲಿ ಬರೆದ ಸಮ್ಮತಿ ರೂಪ

ನಾನು ಮಾಹಿತಿ ಹಾಳೆಯಲ್ಲಿ ನೀಡಲಾಗಿದೆ ಮಾಹಿತಿ ಒದಲು, ಮತ್ತು ಸಂಪೂರ್ಣವಾಗಿ ಅಲ್ಲಿನ ವಿವರಗಳು ಅರ್ಥ. ನಾನು ಪ್ರಾಥಮಿಕ ಪರೀಕ್ಷಕ ಅಥವಾ ತನ್ನ ತಕ್ಕಂತೆ ಅಧಿಕೃತ ಪ್ರತಿನಿಧಿ ಪೂರ್ಣ ವಿವರಗಳನ್ನು ವಿವರಿಸಬಹುದು, ಮತ್ತು ಸಂಪೂರ್ಣವಾಗಿ ಪೂರ್ಣ ಅರ್ಥದಲ್ಲಿ / ಅರಿವು ನನ್ನ ಸ್ವಂತ ಇಚ್ಛೆ, ಪತ್ರದಲ್ಲಿ ನನ್ನ ತೃಪ್ತಿ ನಂತರ ಬೇಕಾದ ಎಲ್ಲಾ ಸ್ಕ್ರೀನಿಂಗ್ ವಿಧಾನಗಳು ಸ್ವಯಂ ನನ್ನ ಒಪ್ಪಿಗೆ ನೀಡಲು ಕಾರಣ ಫಿಲಾರಿಯಾಸಿಸ್ ಗೆ ಸೋಂಕು ಸ್ಕ್ರೀನಿಂಗ್. ನಾನು ಸೋಂಕು ತೆರವುಗೊಳಿಸಲು ಮಾದಕ ಚಿಕಿತ್ಸೆ ನನ್ನ ಒಪ್ಪಿಗೆ ನೀಡಿ.

ನಾನು ಅಧ್ಯಯನದಿಂದ ನನ್ನ ಹಿಂದಕ್ಕೆ ನನ್ನ ಬಲ ಸಂಪೂರ್ಣ ಅರಿವಿದೆ, ಮತ್ತು ಸಂದರ್ಭದಲ್ಲಿ ನಾನು ರೋಗ ನೀಡಲಾಗುವುದು ಎಂದು ಚಿಕಿತ್ಸೆ ಅಥವಾ ಸೂಕ್ತ ಚಿಕಿತ್ಸೆಗಾಗಿ ಕರೆಯಲಾಗುತ್ತದೆ ಭಾಗವಹಿಸುವ ನಿಲ್ಲಿಸಬೇಕು.

ವರ್ಷದ ಈ ದಿನ ನೀಡಲಾಗಿದೆ .... ತಿಂಗಳಲ್ಲಿ in ....

ವ್ಯಕ್ತಿಯ ನನ್ನ ಉಪಸ್ಥಿತಿ ಸಹಿ ಸೈನ್ ಇನ್
ಸಾಕ್ಷಿಯ ಸಹಿ
1
ವಿಳಾಸ ವಿಳಾಸ
....
....
....
2
ವಿಳಾಸ
……………………………………………………………………………………...........
………………………………………………………………………………………..

ಮಾಹಿತಿದಾರ ಅವರಿಗೆ ಮಾಹಿತಿ ಸಹಿ ಹೆಸರು

Appendix 4. INFORMATION SHEET AND CONSENT FORM
TRIPLE DRUG THERAPY FOR LYMPHATIC FILARIASIS STUDY

Person ID (Barcode):

ಭಾಗ ಸಿ

ವಯಸ್ಸು ತರಗತಿಯ ಮಕ್ಕಳು ಫಾರ್ಮ್ ಒಪ್ಪಿಗೆ 7-17 ವರ್ಷಗಳ

ಸಂಶೋಧಕರು: ಡಾ ಪಿ ಜಮ್ಬುಲಿನ್ಗಂ

ನಾವು ಚಿಕಿತ್ಸೆ ಮತ್ತು ನಿಮ್ಮ ಪ್ರದೇಶದಲ್ಲಿ ದುಗ್ದನಾಳ ಫಿಲಾರಿಯಾಸಿಸ್ ತಡೆಗಟ್ಟಲು ಪರಿಣಾಮಕಾರಿ ಔಷಧ ಕಟ್ಟುಪಾಡು ಗುರುತಿಸಲು ಸಂಶೋಧನಾ ಅಧ್ಯಯನ ಮಾಡುತ್ತಿದ್ದಾರೆ. ಔಷಧ ಆಡಳಿತ ಅರ್ಹರಾಗಿರುತ್ತಾರೆ ಅವರೆಲ್ಲರನ್ನೂ ಈ ಅಧ್ಯಯನದಲ್ಲಿ ಭಾಗಿಯಾಗಬಹುದು. ನೀವು ಈ ಅಧ್ಯಯನದ ಭಾಗವಾಗಿ ಎಂದು ಬಯಸುವ ನಿರ್ಧರಿಸಿದ್ದರೆ, ನಾವು ನಿಮಗಾಗಿ ಇವೆ ಚಿಕಿತ್ಸೆಗಳು ಇತರ ರೀತಿಯ ಏನು ಹೇಳುತ್ತವೆ.

ದಾಖಲೆ ಭಾಗ ಒಂದು ನೀಡಲಾಗಿದೆ ಈ ಅಧ್ಯಯನದಲ್ಲಿ ನೀವು ತಿಳಿದುಕೊಳ್ಳಲೇಬೇಕಾದ ಮತ್ತು ಸಂಬಂಧಿತ ಮಾಹಿತಿ ಬಗ್ಗೆ ಕೆಲವು ವಿಷಯಗಳನ್ನು. ಈ ಅಧ್ಯಯನದಲ್ಲಿ ಪಾಲ್ಗೊಳ್ಳುವ ಮೂಲಕ ನೀವು ಹೊಸ ಸೋಂಕನ್ನು ತಡೆಗಟ್ಟುವ ಸೋಂಕಿತ ಅಥವಾ ಪರೋಕ್ಷವಾಗಿ ವೇಳೆ ಸೋಂಕಿನ ಸಂಪೂರ್ಣ ಹೊರಬರಬೇಕಾದರೆ ಸಿಲುಕುವ ನೇರವಾಗಿ ಉಪಯೋಗವಾಗುತ್ತದೆ.

ನಾವು ಈ ಅಧ್ಯಯನದ ಪೂರ್ಣಗೊಳಿಸಿದಾಗ ನಾವು ಕಲಿತದ್ದು ಬಗ್ಗೆ ವರದಿ ಬರೆಯಲು ಮತ್ತು ಈ ವರದಿಯನ್ನು ನಿಮ್ಮ ಹೆಸರು ಒಳಗೊಳ್ಳುವುದಿಲ್ಲ ಅಥವಾ ನೀವು ಅಧ್ಯಯನದಲ್ಲಿ ಎಂದು ಕಾಣಿಸುತ್ತದೆ.

ನಿಮ್ಮ ಭಾಗವಹಿಸುವಿಕೆ ವೈಯಕ್ತಿಕವಾಗಿದ್ದು. ನೀವು ಅಧ್ಯಯನದ ಯಾವುದೇ ಹಂತದಲ್ಲಿ ನಿಮ್ಮ ಭಾಗವಹಿಸುವಿಕೆ ಬೈಠಕ್ ಆಯ್ಕೆ ಮಾಡಬಹುದು. ನಿಮ್ಮ ಪೋಷಕರು ತುಂಬಾ ಅಧ್ಯಯನ ಬಗ್ಗೆ ಮತ್ತು ನಿಮ್ಮ ಭಾಗವಹಿಸುವಿಕೆ ಒಪ್ಪಿಗೆ ಅವರಿಂದ ಪಡೆಯಲಾಗುವುದು.
ನೀವು ಈ ಅಧ್ಯಯನದಲ್ಲಿ ಬಯಸುತ್ತೇನೆ ನಿರ್ಧರಿಸಿದ್ದರೆ, ಸಹಿಮಾಡಿ ಮಾಡಿ.

ನಾನು ___________________________, ಈ ಸಂಶೋಧನಾ ಅಧ್ಯಯನದಲ್ಲಿ ಬಯಸುತ್ತೇನೆ.

______________________________ _____

(ದಿನಾಂಕ) (ಇಲ್ಲಿ ನಿಮ್ಮ ಹೆಸರನ್ನು ಸೈನ್)

Appendix 4. INFORMATION SHEET AND CONSENT FORM
TRIPLE DRUG THERAPY FOR LYMPHATIC FILARIASIS STUDY

Person ID (Barcode):

ಪಾರ್ಟ್ ಡಿ

ಪೋಷಕ ಮಾಹಿತಿಯುಕ್ತ ಸಮ್ಮತಿಯ ನಮೂನೆ

ನಿಮ್ಮ ಮಗುವಿನ ಪ್ರಸ್ತಾವಿತ ಪರಿಶೋಧಕ ಅಧ್ಯಯನದ ಸೇರಲು ಆಮಂತ್ರಿಸಲಾಗಿದೆ ಭಾಗ ಎ ನಿಮ್ಮ ಮಗುವಿನ ಸೇರಲು ಅವಕಾಶ, ಅಥವಾ ಸೇರಲು ನಿರ್ಧಾರ ವಿವರಿಸಿದರು, ಸಂಪೂರ್ಣವಾಗಿ ವೈಯಕ್ತಿಕವಾಗಿದ್ದು.

ಈ ಸಂಶೋಧನಾ ಅಧ್ಯಯನದಲ್ಲಿ, ನಾವು ದುಗ್ಧನಾಳ ಫಿಲಾರಿಯಾಸಿಸ್ ತೊಡೆದುಹಾಕಲು ದಕ್ಷತೆ ಮತ್ತು ಹೊಸ ಔಷಧ ಕಟ್ಟುಪಾಡು ಸುರಕ್ಷತೆ ತನಿಖೆ ಮಾಡುತ್ತಿದ್ದೇವೆ.

ಅಧ್ಯಯನದಲ್ಲಿ ಮಕ್ಕಳ ಭಾಗವಹಿಸುವಿಕೆ ಲಿಖಿತ ಅನುಮತಿ ರೂಪ
ನಾನು ಮಾಹಿತಿ ಹಾಳೆಯಲ್ಲಿ ನೀಡಲಾಗಿದೆ ಮಾಹಿತಿ ಓದಲು, ಮತ್ತು ಸಂಪೂರ್ಣವಾಗಿ ಅಲ್ಲಿನ ವಿವರಗಳು ಅರ್ಥ. ನಾನು ಪೂರ್ಣ ಅರ್ಥದಲ್ಲಿ / ಅರಿವು ನನ್ನ ಸ್ವಂತ ಇಚ್ಛೆ, ಪತ್ರದಲ್ಲಿ ಪೂರ್ಣ ವಿವರಗಳು ಪ್ರಮುಖ ಸಂಶೋಧಕ / ತಕ್ಕಂತೆ ಅಧಿಕೃತ ಪ್ರತಿನಿಧಿ ವಿವರಿಸಬಹುದು, ಮತ್ತು ಸಂಪೂರ್ಣ ತೃಪ್ತಿ ಹೊಂದಿರುವ ನಾನು, ನನ್ನ ಒಪ್ಪಿಗೆ ಎಲ್ಲಾ ಸ್ಕ್ರೀನಿಂಗ್ ವಿಧಾನಗಳು / ನನ್ನ ಮಗಳು ನನ್ನ ಮಗ ನೀಡಲು ಕಾರಣ ಫಿಲಾರಿಯಾಸಿಸ್ ಸೋಂಕು ಸ್ಕ್ರೀನಿಂಗ್ ಅಗತ್ಯವಿದೆ. ನಾನು ಸೋಂಕಿನ ನಿರ್ಮೂಲ ಔಷಧಿಗಳನ್ನು ನನ್ನ ಮಗು ಚಿಕಿತ್ಸೆ ನನ್ನ ಒಪ್ಪಿಗೆ ನೀಡಿ.

ನಾನು ಅಧ್ಯಯನದಿಂದ ನನ್ನ ವಾರ್ಡ್ ಹಿಂದಕ್ಕೆ ನನ್ನ ಬಲ ಸಂಪೂರ್ಣ ಅರಿವಿದೆ, ಮತ್ತು ಸಂದರ್ಭದಲ್ಲಿ ನನ್ನ ವಾರ್ಡ್ ರೋಗ ನೀಡಲಾಗುವುದು ಎಂದು ಚಿಕಿತ್ಸೆ ಅಥವಾ ಸೂಕ್ತ ಚಿಕಿತ್ಸೆಗಾಗಿ ಕರೆಯಲಾಗುತ್ತದೆ ಭಾಗವಹಿಸುವ ನನ್ನ ಮಗು ನಿಲ್ಲಿಸಬೇಕು.

ರಿಸರ್ಚ್ ಭಾಗವಹಿಸಲು ಒಂದು ಶಿಶು ಅನುಮತಿ

ಪೋಷಕರು ಅಥವಾ ಕಾನೂನು ಪೋಷಕನಾಗಿ, ಈ ಸ್ವರೂಪದಲ್ಲಿ ವರ್ಣಿಸಲಾಗಿದೆ ಸಂಶೋಧನಾ ಅಧ್ಯಯನದಲ್ಲಿ ಪಾಲ್ಗೊಂಡಿರುವ ಆಗಲು ಅಧಿಕಾರ __________________________ (ಮಗುವಿನ ಹೆಸರು).

ಜನ್ಮ ಮಗುವಿನ ದಿನಾಂಕ: __________________
ಹೆತ್ತವರು ಅಥವಾ ಕಾನೂನುಬದ್ಧ ಪೋಷಕರಿಗೆ ಸಹಿ ದಿನಾಂಕ

ನನ್ನ ಉಪಸ್ಥಿತಿಯಲ್ಲಿ ಸಹಿ:
ಸಾಕ್ಷಿಯ ಸಹಿ: ವಿಟ್ನೆಸ್ 1 ಸಹಿ: 2
ವಿಳಾಸ ವಿಳಾಸ

....
....

ಮಾಹಿತಿದಾರ ಅವರಿಗೆ ಮಾಹಿತಿ ಸಹಿ ಹೆಸರು

Appendix 5: PARTICIPANT ENROLLMENT FORM

TRIPLE DRUG THERAPY FOR LYMPHATIC FILARIASIS STUDY

PERSON ID (Barcode):

1. STUDY SITE INFORMATION

Team:	Person conducting enrollment:
Enrollment Date (DD-MM-YYYY):	
Consent Method (**required**)<br>☐Self<br>☐Parent<br>☐Other guardian (specify):<br>☐No consent (**do not continue**)	Enrollment Location Type<br>☐Home
Enrollment Village:	
Treatment arm: ☐ DA ☐IDA	

2. PERSONAL INFORMATION AND MEDICAL HISTORY

Surname:		First Name:
Gender: ☐M ☐F	Age: Years	Village of Residence:
Study ID (Barcode):<br>(also affix barcode at the top of each page of the form)		
Females only: When was the date of our last menstrual period?<br>☐Definitely less than 4 weeks ago<br>☐More than 4 weeks ago (**exclude**)<br>☐ Post-menopause<br>☐Uncertain (**exclude**)		
Males only: Do you have swelling/enlargement of your scrotum?☐Yes ☐No		
Males only: Do you have pain in testicles or scrotum? ☐Yes ☐No		
Do you have any of the following chronic medical conditions?<br>☐Hypertension (high blood pressure)<br>☐Asthma or chronic lung disease **(exclude)**<br>☐Chronic kidney disease (renal insufficiency) **(exclude)**<br>☐Severe chronic liver disease **(exclude)**<br>☐Diabetes<br>☐Other (specify): **(exclude if is severe enough to interfere with daily living)**		
Do you have swelling in your arms or legs (lymphedema)? ☐Yes ☐No		
If lymphedema: Indicate which limbs are affected and with what grade.<br>Grading scale:<br>0. No lymphedema<br>1. Mild, reversible with elevation (not present when first arising from sleep)<br>2. Not reversible with elevation (present when arising from sleep) but no folds<br>3. Shallow skin folds (can see base of fold with Participant movement of affected limb/joint)<br>4. Knobs<br>5. Deep skin folds (cannot see base of fold with movement of affected limb/joint)<br>6. Mossy lesions		

Appendix 5: PARTICIPANT ENROLLMENT FORM

TRIPLE DRUG THERAPY FOR LYMPHATIC FILARIASIS STUDY

PERSON ID (Barcode):

Left arm	☐0	☐1	☐2	☐3	☐4	☐5	☐6
Left leg	☐0	☐1	☐2	☐3	☐4	☐5	☐6
Right arm	☐0	☐1	☐2	☐3	☐4	☐5	☐6
Right leg	☐0	☐1	☐2	☐3	☐4	☐5	☐6

Did you use a bed net last night? ☐Yes ☐No

Does your house have screens on the windows? ☐Yes ☐No

Do you spray indoors to prevent mosquitoes? ☐Yes ☐No

Did you swallow medicines during MDA treatment for filariasis in the last year?

- ☐Yes, If YES enter the date (MM-YYYY):____________ ☐ Do not remember date
- ☐No
- ☐Uncertain
- ☐Drug NOT distributed

Have you ever taken any of the following medications called?

DEC 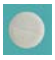 ☐ Yes ☐ No (explain) ☐ Don't know

Albendazole 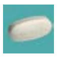 ☐ Yes ☐ No explain) ☐ Don't know

Ivermectin 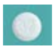 ☐ Yes ☐ No (explain) ☐ Don't know

3. PRE-TREATMENT AE ASSESSMENT

Do you feel well today? ☐Yes ☐No

If unwell, are you too sick to do your normal daily activities?

- ☐Yes (**exclude**)
- ☐No (continue)

Which of the following symptoms have you experienced in the past 2 days?

Fever	☐Yes ☐No
Headache	☐Yes ☐No
Nausea	☐Yes ☐No
Vomiting	☐Yes ☐No
Diarrhea	☐Yes ☐No
Abdominal pain	☐Yes ☐No
Acute swelling, beyond baseline lymphedema	☐Yes ☐No
Specify Location: ☐Upper limb ☐Lower limb ☐Breast ☐Scrotum	
Joint or muscle pain	☐Yes ☐No
Swollen or painful nodes (armpit or groin)	☐Yes ☐No
Men only: testicular or scrotal pain	☐Yes ☐No
Rash (specify location):______________________________	☐Yes ☐No
Is the rash in spaces between your fingers?	☐Yes ☐No
Itching skin	☐Yes ☐No
Cough	☐Yes ☐No
Difficulty breathing (wheezing or dyspnea)	☐Yes ☐No

Appendix 5: PARTICIPANT ENROLLMENT FORM

TRIPLE DRUG THERAPY FOR LYMPHATIC FILARIASIS STUDY

PERSON ID (Barcode):

Fatigue	☐Yes ☐No
Dizziness, giddiness, or fainting	☐Yes ☐No
Confusion or excess drowsiness	☐Yes ☐No
Other illness or symptoms (specify):_____________________	☐Yes ☐No
Have you ever suffered from scabies?<br>☐Yes<br>☐No<br>☐Don't know	
Additional notes or comments:	

4. EXAMINATION

Team (**required**):	Clinician (**required**):
Data Entry Clerk ID (**required**):	

**Measurements**	**Values / status**
Height (cm)	
Weight (kg)	
BMI (calculated)	
Scabies	☐Yes ☐No
If Yes, please take photograph	

5. PRE MDA BLOOD EXAMINATION RESULTS

Test type	Aliquot Number	Result
Antigen (FTS)		
Mf count		

Appendix 6: PARTICIPANT MONITORING FORM

TRIPLE DRUG THERAPY FOR LYMPHATIC FILARIASIS STUDY

PERSON ID (Barcode):

1. PERSONAL INFORMATION

Surname:		First Name:	
Gender: ☐M ☐F	Age: Years	Village of Residence:	
Treatment Village:		Treatment Date: (DD-MM-YYYY)	

2. ASSESSMENT INFORMATION

Day 1 and 2: How have you been feeling since taking the tablets?

Day 1

☐Well, no complaints (*no further assessment needed)*

☐Otherwise (*please complete adverse event table for day 1*)

Day 2

☐Well, no complaints (*no further assessment needed)*

☐Otherwise (*please complete adverse event table for days 1-2*)

Days 3-7: record any symptoms the Participant is reporting in the treatment effect table

**Table 1: Reported Symptoms**								
Record a symptom grade from 0-5 for each day on which the Participant experienced symptoms. For subjects with no complaints on day 1 and 2 and who do not report with complaints during passive follow-up (days 3-7), leave this table blank. For subjects reporting ANY symptom on day 1-2 or presenting with any complaint on day 3-7, please ask about EACH listed symptom, not just the symptom the subject is reporting. Refer to the Appendix 6a for symptom-specific scoring criteria. Subjects reporting symptoms marked with an asterisk (*) should be examined for physical findings and have their blood pressure and temperature measured and recorded in Table 2.								
	**Post-treatment day(s) on which symptoms or signs were present**							
**Symptoms/Signs**	**Day 0**	**Day 1**	**Day 2**	**Day 3**	**Day 4**	**Day 5**	**Day 6**	**Day 7**
Fever*								
Headache								
Nausea								
Vomiting								
Diarrhea								
Abdominal pain								
Acute swelling, beyond baseline lymphedema (specify location below)								
Upper limb								
Lower limb								
Breast								
Scrotum								
Joint or muscle pain								
Swollen or painful nodes (armpit or groin)*								

Appendix 6: PARTICIPANT MONITORING FORM

TRIPLE DRUG THERAPY FOR LYMPHATIC FILARIASIS STUDY

PERSON ID (Barcode):

Men only: testicular or scrotal pain								
Rash (specify location and brief description)*:								
Itching skin								
Cough								
Difficulty breathing (wheezing or dyspnea)*								
Fatigue								
Dizziness, giddiness, or fainting*								
Confusion or excess drowsiness*								
Other illness or symptoms (specify):								

If there is any symptom grade > 2, you must notify the supervising medical officer and the subject must be evaluated by the medical team.

**Table 2: Physical Examination**								
You must complete this table for any subject reporting any of the symptoms marked with an asterisk (*) in Table 1. Record the requested information in the appropriate space under each post-treatment day on which an assessment was made.								
	**Post-treatment day(s)**							
**Measurements**	**Day 0**	**Day 1**	**Day 2**	**Day 3**	**Day 4**	**Day 5**	**Day 6**	**Day 7**
Temperature								
Blood pressure, sitting								
Blood pressure, recumbent (*measure only if sitting systolic BP <90)*								
**Post-Exam Adverse Event Grade** (Assign grade of 0-5 for the adverse reactions below based on physical exam. See Appendix 6a under "post-exam assessment" for specific grading criteria)								
Allergic reaction (e.g., rash, urticaria, bronchospasm)								
Hypotension (low blood pressure)								
Lymphangitis (streaks of redness, warmth, and swelling in arms or legs)								

Appendix 6a.Guide for assigning adverse event severity grades 1-4
(G*rade 0 = no symptoms; grade 5 = death from adverse event*)

Symptoms/Signs	Grades			
	1. Mild	2. Moderate	3. Severe	4. Life-threatening
Fever (non-axillary temperatures only)	38.0 – 39.0ºC	39.1 – 40.0ºC	> 40.0ºC	> 40.0ºC for > 48 hrs
Headache	Mild pain not interfering with work or school	Moderate pain; pain or analgesics interfering with ability to work or attend school	Severe pain; pain or analgesics interfering with activities of daily living	Disabling, duration > 48 hr
Nausea	Able to eat	Oral intake significantly decreased	No significant intake, requiring IV fluids	-
Vomiting	1 episode in 24 hours over pretreatment	2-5 episodes in 24 hours over pretreatment	≥ 6 episodes in 24hours, or need for IV fluids (0uParticipant)	Hemodynamic collapse or overnight hospitalization
Diarrhea	Increase of < 4 stools/day over pre-treatment	Increase of 4-6 stools/ day, or nocturnal stools	Increase of ≥ 7 stools/ day or need for outParticipant parenteral support for dehydration	Physiologic consequences wth hemodynamic collapse or requiring hospitalization
Abdominal pain	Mild pain not interfering with work or school	Moderate pain; pain or analgesics interfering with ability to work or attend school	Severe pain; pain or analgesics interfering with activities of daily living	Disabling, duration > 48 hr
Acute swelling (beyond baseline lymphedema)	Mild, not interfering with work or school	Moderate, unable to work or attend school 1 day	Severe, unable to work/school >1 day	Severe, limiting activities of daily living (unable to walk) > 2 days
Joint or muscle pain	Mild pain not interfering with work or school	Moderate pain; pain or analgesics interfering with ability to work or attend school	Severe pain; pain or analgesics interfering with activities of daily living	Disabling, duration > 48 hr
Swollen or painful nodes (armpit or groin)*	Mild, not interfering with work or school	Moderate, unable to work or attend school 1 day	Severe, unable to work/school >1 day	Severe, limiting activities of daily living (unable to walk) > 2 days
Men only: testicular or scrotal pain	Mild, not interfering with work or school	Moderate, unable to work or attend school 1 day	Severe, unable to work/school >1 day	Severe, limiting activities of daily living (unable to walk) > 2 days

Appendix 6a.Guide for assigning adverse event severity grades 1-4

(G*rade 0 = no symptoms; grade 5 = death from adverse event*)

Rash	Localized rash (covers only one part of the body)	Diffuse rash (covers multiple parts of the body)	Diffuse rash (covers multiple parts of the body) AND has any blisters or ulcers or mouth sores	Extensive areas with blisters or ulcers OR peeling or blackening of skin
Itching skin	Mild, not interfering with work or school	Moderate, unable to work or attend school 1 day	Severe, unable to work/school >1 day	
Cough	Mild, relieved by non-prescription medication	Requiring narcotic antitussive	Severe cough or coughing spasms, poorly controlled by treatment	Hospitalization or respiratory failure requiring mechanical ventilation
Difficulty breathing (wheezing or dyspnea)	Mild, not interfering with work or school	Moderate, unable to work or attend school for 1 day	Severe, more than 1 day and required transfer to clinic or hospital	Hospitalization or respiratory failure requiring mechanical ventilation
Fatigue	Mild, not interfering with work or school	Moderate, unable to work or attend school at least 1 day	Unable to perform activities of daily living, > 1day	Required hospitalization
Dizziness, giddiness, or fainting	Mild, not interfering with work or school	Moderate, unable to work or attend school for 1 day, but no fainting	Any loss of consciousness (fainting)	-
Confusion or excess drowsiness*	Mild, not interfering with work or school	Moderate; confusion or drowsiness interfering with ability to work	Confusion, loss of memory, or sleepiness interfering with activities of daily living	Delerium, inability rouse, or coma
Other illness or symptoms	Mild, not interfering with work or school	Moderate, unable to work or attend school at least 1 day	Unable to perform activities of daily living, > 1day	Required hospitalization

Appendix 6a.Guide for assigning adverse event severity grades 1-4
(G*rade 0 = no symptoms; grade 5 = death from adverse event*)

Post-Exam Assessment	Grades			
	**1. Mild**	**2. Moderate**	**3. Severe**	**4. Life-threatening**
Acute allergic reaction	Transient rash, drug Fever <38ºC (<100.4ºF)	Urticaria, drug fever ≥38ºC (≥100.4ºF) and/or asymptomatic bronchospasm	Symptomatic bronchospasm, requiring parenteral medication(s) with or without urticaria	Anaphylaxis with hypotension required hospitalization
Hypotension (low blood pressure)	Changes, but not requiring therapy (including transient orthostatic hypotension)	Requiring brief fluid replacement (such as oral rehydration) but not hospitalization	Requiring i.v. fluids without overnight hospitalization. No sequelae.	Required overnight hospitalization for i.v. fluids, or Shock (acidemia and impaired vital organ function due to tissue hypoperfusion)
Lymphangitis	Mild, not interfering with work or school	Moderate, unable to work or attend school 1 day	Severe, unable to work/school >1 day	Severe, limiting activities of daily living (unable to walk) > 2 days

Note on general aspects of grading

0 = No adverse event or within normal limits
1 = Mild adverse event, does not interfere with work or school
2 = Moderate adverse event, interferes with work or school at least 1 day
3 = Severe and undesirable adverse event; interferes with ADL, requires medical assessment
4 = Potentially life-threatening or disabling adverse event; requires transfer to medical facility
5 = Death

Note: *Any event > grade 2 requires a medical evaluation and notification of the medical officer. Any grade 4 or 5 event or overnight hospitalization requires a Serious Adverse Event Report, see Appendix 6*

Appendix 7: ADVERSE EVENT EVALUATION AND REPORT FORM (AEERF)
TRIPLE DRUG THERAPY FOR LYMPHATIC FILARIASIS STUDY

PERSON ID (Barcode):

Instructions: Complete this form AFTER completing the Participant Monitoring Form for anyone with symptoms or signs of <u>grade 3 or higher</u> *(unable to perform activities of daily living without assistance for at least one day). The purpose of this form is to provide additional information on more severe adverse events and to assist the medical officer in determining whether a Serious Adverse Event (SAE) has occurred. Please refer to page 65 of Appendix 7a for definitions.*

1. PERSONAL INFORMATION

<table>
<tr><td colspan="2">Surname:</td><td colspan="2">First Name:</td></tr>
<tr><td>Gender: ☐M ☐F</td><td>Age:_____Years</td><td>Weight:_____Kg</td><td>Height:_____cm</td></tr>
<tr><td colspan="4">Village of Residence:</td></tr>
</table>

2. MDA TREATMENT

<table>
<tr><td>Treatment Date (DD-MM-YYY)___________
Treatment Village:______________________
Anything irregular about treatment?
☐No ☐Yes (specify):</td><td>Medications received
☐ Albendazole (dose:____mg)
☐ DEC (dose:____mg)
☐ Ivermectin (dose:____mg)</td></tr>
<tr><td colspan="2">Was this the first treatment with any of the MDA medications? If No, explain when and circumstances of prior treatment.
Albendazole ☐Yes ☐No (explain):
DEC ☐Yes ☐No (explain):
Ivermectin ☐Yes ☐No (explain):</td></tr>
</table>

3. OTHER MEDICATIONS AT TIME OF MDA

Please include prescription and non-prescription medications/supplements/herbal remedies taken within 5 days of the MDA. DO NOT include medications used to treat the SAE.

Medication	Indication	Dose and Frequency	Days on which each medication was taken, relative to MDA (if taken the day of MDA, mark “0”; the day before, mark “-1”; the day after, “+1”, and so forth.)
			-5 -4 -3 -2 -1 0 +1 +2 +3 +4 +5 uncertain
			-5 -4 -3 -2 -1 0 +1 +2 +3 +4 +5 uncertain
			-5 -4 -3 -2 -1 0 +1 +2 +3 +4 +5 uncertain
			-5 -4 -3 -2 -1 0 +1 +2 +3 +4 +5 uncertain

Appendix 7: ADVERSE EVENT EVALUATION AND REPORT FORM (AEERF)
TRIPLE DRUG THERAPY FOR LYMPHATIC FILARIASIS STUDY

PERSON ID (Barcode):

4. DESCRIPTION OF THE ADVERSE EVENT

<table>
<tr><td>Date of onset (DD-MM-YYYY):</td><td>How long after drugs were taken?
_____hours OR _____days</td></tr>
<tr><td colspan="2">Clinical signs and symptoms (please describe)

Do you think this adverse event is/was life-threatening? ☐Yes ☐No</td></tr>
<tr><td colspan="2">Was the Participant hospitalized? ☐Yes ☐No
If yes, indicate
1. Date of admission (DD-MM-YYYY):

2. Reason for admission:

3. Date of discharge (DD-MM-YYYY):

4. Clinical course, including drug treatments given to treat adverse event:

Attach a copy of any medical records relating to the diagnosis and treatment of the adverse event</td></tr>
<tr><td colspan="2">Laboratory results and diagnostic tests (indicate date, test name, and results):

</td></tr>
</table>

Appendix 7: ADVERSE EVENT EVALUATION AND REPORT FORM (AEERF)

TRIPLE DRUG THERAPY FOR LYMPHATIC FILARIASIS STUDY

PERSON ID (Barcode):

5. **ADVERSE EVENT OUTCOME (Check only ONE)**

☐Recovering/resolving		
☐Not recovered/not resolved		
☐Recovered/resolved	Date: (DD-MM-YYY)	
☐Recovered/resolved with sequelae	Date:(DD-MM-YYY)	Sequelae:
☐Unknown		
☐Fatal(death)	Date:(DD-MM-YYY)<br>Autopsy: ☐Not done ☐Done (*provide report*) ☐Planned ☐ Status Unknown<br>Death certificate: ☐Provided ☐Requested ☐Not available ☐ Status Unknown	

Appendix 7: ADVERSE EVENT EVALUATION AND REPORT FORM (AEERF)
TRIPLE DRUG THERAPY FOR LYMPHATIC FILARIASIS STUDY

PERSON ID (Barcode):

6. CONCLUSIONS (to be completed by the health-care provider)

Presumptive diagnosis:
Do you think this adverse event was caused by the MDA medications? *Refer to Appendix 7a for detailed explanation of choices.*<br>☐ Definitely<br>☐ Probably (explain):<br>☐ Possibly (explain):<br>☐ Unrelated<br>If "unrelated", what do you believe was the cause of the adverse event?
Does this event meet the criteria for a Serious Adverse Event (SAE*)? Refer to Appendix 7a for detailed definitions of criteria.*<br>☐ Yes, based on the following criteria<br>☐ Death<br>☐ Life-threatening<br>☐ Hospitalization<br>☐ Disability or permanent damage<br>☐ Other serious important medical event: specify<br>☐ No

REPORTER INFORMATION AND SIGNATURES

Investigator Name:	Investigator Signature:	Date:
Reporter Name:	Reporter Signature:	Date:
Reporter's phone number:	Reporter's email address:	

Appendix 7a: Instructions for Reporting Severe Adverse Events

An Adverse Event Evaluation and Report Form (AEERF) should be completed for every severe adverse event (those scoring grade 3 or higher, see appendix 6). However, a grade 3 or **severe** adverse event is NOT the same as a **Serious** Adverse Events (**SAE**) and the majority of grade 3 adverse events will not be classified as SAE. SAE is a regulatory term describing any untoward medical occurrence with any of the following characteristics:

- Results in death
- Requires in Patient hospitalization
- Results in permanent or significant disability
- Is life-threatening
- Results in a congenital abnormality or birth defect
- Results in other serious important medical events

The AEERF should guide the medical officer or health care provider evaluating the Participant experiencing a severe AE to determine whether an SAE has occurred. All SAE must be reported promptly.

Required Reporting

A written report or case report form (CRF—in this study, the AEERF) must be faxed or sent by email (scanned records) in the stated timeframes to the Project Coordinator and Dr. Rashmi Arora at ICMR for the events listed below. **Copies of these reports will be sent to the Medical Monitor at the same time.**

- **Unexpected Fatal or Life Threatening events with any possible association with use of test article or participation in the study must be reported within <u>*7 calendar days.*</u>**
- **Events that are both Serious and Unexpected with any possible association with the use of the test article or study participation must be reported within <u>*15 calendar days.*</u>**

Guidelines for Reporting

Standard Reporting Information

The following information should be included in the report/CRF (additional information may be requested):

- <u>Description of the event</u>

Date, time of onset

Clinical history
Associated signs and symptoms
Temporal association with study agent
Medical management, including rationale
Pertinent laboratory tests
Severity – *see definitions or toxicity score*
Causal relationship to the study drug/vaccine

- <u>Other information</u>

Appendix 7a: Instructions for Reporting Severe Adverse Events

Relevant past medical history

Concomitant medications
Autopsy report or expectation of an autopsy in the case of death

- Outcome of event

Date, time of resolution, if resolved

- Plans for study subject

Follow-up

Treatment of event
Return to treatment/Contraindicate

- Location/Study Center

- Reporting Physician

- Verification of notification to IRB and Safety Monitor or DSMB

Definitions

- Adverse Event [Experience] (AE):
  Any untoward medical occurrence, including dosing errors, that may arise during administration of study agent, and which may or may not have a causal relationship with the study agent.

- Unexpected Adverse Event [Experience]:
  Any adverse experience that has not been previously observed (i.e., included in the labelling), whether or not the event is anticipated because of the pharmacologic properties of the study agent.

- Serious Adverse Experience (SAE):
  Any adverse experience occurring at any dose that results in any of the following outcomes:

  a. Death
  b. Life threatening – defined as an experience that places the Participant or subject, in the view of the Investigator, at *immediate risk* of death from the reaction as it occurred. (Note; this does not include a reaction that, had it occurred in a more severe form, might have caused death.)
  c. Requires in patient hospitalization or prolongation of existing hospitalization
  d. Results in a congenital anomaly or birth defect
  e. Results in a persistent or significant disability or incapacity
  f. Important medical events that may not result in death, be life-threatening, or require hospitalization may be considered a serious adverse experience when, based upon appropriate medical judgment, they may jeopardize the Participant or subject and may require medical or surgical intervention to prevent one of the outcomes listed in this definition. *(the event might be defined as serious based on progression of grade if Toxicity Tables are being used.)*

Appendix 7a: Instructions for Reporting Severe Adverse Events

- Severity

Adverse experience/events should be assessed by the on-site investigator as to their severity and/or intensity.

a. Life threatening
b. Severe: incapacitating with inability to work or do usual activity
c. Moderate: enough discomfort to cause interference with usual activity
d. Mild: awareness of sign or symptom, but easily tolerated

Relationship or Association with Use of Study Agent or Participation in the Study

Causal relationship with the investigational study treatment must be assessed by the on-site investigator using the following or similar terms:

- **Definite** – clear-cut temporal association, with a positive re-challenge test or laboratory confirmation.
- **Probable** – clear-cut temporal association, with improvement upon drug withdrawal, and not reasonably explained by the subject’s known clinical state.
- **Possible** – less clear temporal association; other aetiologies are possible.
- **None** – no temporal association with the study drug; related to other aetiologies such as concomitant medications or conditions, or subject’s known clinical state.

Other Reporting obligations

- For all studies, there must be compliance with the clinical site IRB’s policy for reporting adverse events.

Appendix 8: Medical Monitoring activities

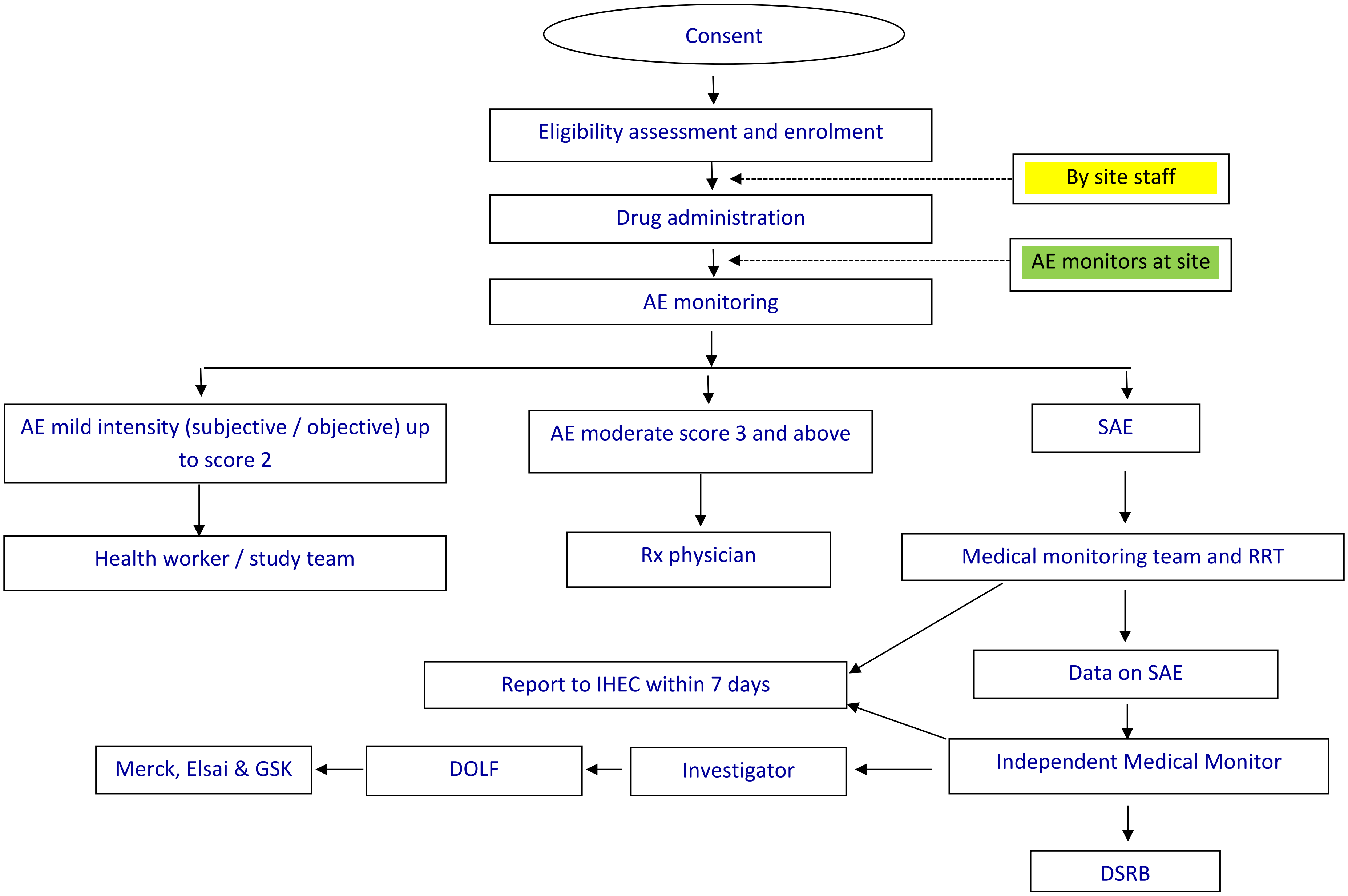

Appendix 9: Guidelines for Treatment acceptability

A mixed method approach, combining the use of a community survey, focus group discussions and in depth interviews with key informants will be followed. A composite score will measure acceptability, combining outcomes like the respondents' intention to take the treatment again and willingness to recommend it to other family members. Acceptability will be analyzed by the impact of some of the known factors that impact compliance: perception of AE, knowledge about AE, perceptions about the drug characteristics (safe, number of pills, taste), knowledge of vector, belief that the treatment is associated with health, and others. In order to assess the difference between the two treatment arms, the sampling frame for the community survey will take into account which regimen the individual received.

To complement the community surveys and provide further in depth analysis, focus group discussions (FGD) are planned with specific groups in the community, namely men, women, young people and community health workers. The FGDs will provide further insight and depth for some of the questions asked in the community survey. Specifically FGDs will investigate issues expected to relate to the 3-drug regimen: number of pills, perception of AE, how to ensure directly observed treatment and proposed messages to encourage compliance.

These results will be further substantiated by interviews with key community leaders, as well as community and professional health workers working in LF elimination at the village level. These interviews will provide an understanding of the macro level issues that key informants perceive as critical to consider with the use of the 3-drug therapy. With this, interview respondents will be asked what advantages and concerns they have with regards to the 3-drug regimen based on their participation in and understanding of the safety trial.

These surveys will be conducted after two weeks after the completion of the drug administration in order to provide some time for the effects of ivermectin to become apparent but no later than one month.

<u>Questionnaire Development for community survey</u>

Acceptability of the 3-drug therapy will be measured in a composite score from the following questions:

- Intention to take LF drugs in the future measured on a 5-point scale ranging from "I will never take this drug again" to "I will definitely take this drug again." (Adapted from Liau and Zimet 2001)
- Willingness to encourage other family members to take the LF drug, if offered in the future measured as a 5-point scale ranging from "I will never encourage my family to take the LF drugs" to "I will definitely encourage my family to take the LF drugs."
- Overall feeling about the LF elimination program as a 5-point scale ranging from "Very negative" to "Very positive"
- Perception of health since taking the LF drugs as a 5-point scale ranging from "Considerably worse" to "greatly improved"
- In addition to the scoring, each outcome can be converted to a binary variable for multivariate modeling.
- Inputs / Exposure variables:

Appendix 9: Guidelines for Treatment acceptability

- SES data
- Data from safety trial (clinical presence of AE, MF rate, household information)
- Treatment arm (2-drug versus 3-drug)
- Informed about the treatment before receiving the drug (e.g. did they receive any information)
- Belief in the efficacy of the treatment to eliminate / prevent LF (e.g. believe that the drugs work to prevent / treat LF)
- Belief in the efficacy of the treatment to treat scabies (e.g. believe that the drugs work to treat scabies)
- Belief in the efficacy of the treatment to treat other intestinal worms (e.g. believe that the drugs work to treat worms)
- Knowledge of the 'positive' component of AE (e.g. occur because the medicine is working)
- Perception of AE (e.g. none, mild, moderate, severe)
- Understanding that taking LF medicine is good for promoting health
- Knowledge that mosquitoes transmit LF
- Perception that the rest of the family/ household would take the LF drugs, if offered in the future (yes/no)
- Belief that the drug distributors are doing a good job (using a 10-point scale)
- Perceptions of the drugs (e.g. safe, neutral, dangerous)
- Components of the drugs (e.g. number, size, taste of pills)
- Emotions surrounding LF treatment (e.g. how does taking LF treatment make you feel?)

Focus Group Discussion

Range of issues to explore include:

- How is LF elimination different / similar from the other health programs in their village?
- What are the health benefits from taking the treatment?
- What are the social benefits from taking the treatment?
- Do people like to take the pills in front of the distributor? Why or why not?
- How do you feel about the number of pills that you have to take?
- Why don't people want to take it?
- Did you have any side effects after you took the drugs (positive or negative)? How did you feel about them?
- What suggestions do you have to promote MDA to their community? Household?
- Are there any specific messages you would recommend to us?

In depth interviews with key informants

<u>Range of issues to explore include</u>:

Appendix 9: Guidelines for Treatment acceptability

- What are the advantages of the 3-drug therapy in MDA? Disadvantages?
- What opportunities do they see in the administration of the 3-drug therapy, versus the 2-drug therapy?
- What concerns or challenges do they see in the administration of the 3-drug therapy, versus the 2-drug therapy?
- How do they feel about the number of pills that the community is asked to take?
- How do they feel about the side effects people might have / have?
- What suggestions do they have to promote MDA in this village? This province? The country? What messages would they recommend using?
- Which groups of people do they think will be difficult to reach with future MDA? Why? Any advice to approach them?
