# Supplementary material for: An open label, block randomized, community study of the safety and efficacy of co-administered ivermectin, diethylcarbamazine plus albendazole vs. diethylcarbamazine plus albendazole for lymphatic filariasis in India
Source: PLoS Negl Trop Dis. 2021 Feb 16;15(2):e0009069. doi: 10.1371/journal.pntd.0009069 (PMC7909694; doi:10.1371/journal.pntd.0009069)
Supplement: S1 Table — (DOCX) [file pntd.0009069.s002.docx]

**S1 Table.** Participant follow-up rates for assessment of adverse events after treatment

| Participant’s availability during | Drug regimen  (IDA: n=4758; DA: n=4160) | Found (%) |
| --- | --- | --- |
| Visit 1 | IDA | 4544 (95.5) |
|  | DA | 4083 (98.1) |
| Visit 2 | IDA | 4534 (95.3) |
|  | DA | 4093 (98.4) |
| Visit 1 & 2 | IDA | 4393 (92.3) |
|  | DA | 4048 (97.3) |
| Visit 1 OR 2 | IDA | 4685 (98.5) |
|  | DA | 4128 (99.2) |

Follow-up rates differ significantly between treatment groups (*P* < 0.001 for all the visits).
