# Supplementary material for: An open label, block randomized, community study of the safety and efficacy of co-administered ivermectin, diethylcarbamazine plus albendazole vs. diethylcarbamazine plus albendazole for lymphatic filariasis in India
Source: PLoS Negl Trop Dis. 2021 Feb 16;15(2):e0009069. doi: 10.1371/journal.pntd.0009069 (PMC7909694; doi:10.1371/journal.pntd.0009069)
Supplement: S2 Table — (DOCX) [file pntd.0009069.s003.docx]

**S2 Table.** Type of adverse events by severity and treatment regimens

| Adverse Events | IDA (total number of AEs= 623) | | | | DA (total number of AEs= 396) | | | |
| --- | --- | --- | --- | --- | --- | --- | --- | --- |
|  | Grade 1 | Grade 2 | Grade 3 | All Grades | Grade 1 | Grade 2 | Grade 3 | All Grades |
| Fever | 132 (21.2) | 11 (1.8) | 0 (0.0) | 143 (23.0) | 52 (13.1) | 1 (0.3) | 0 (0.0) | 53 (13.4) |
| Headache | 124 (19.9) | 14 (2.2) | 0 (0.0) | 138 (22.2) | 86 (21.7) | 2 (0.6) | 0 (0.0) | 88 (22.2) |
| Dizziness | 65 (10.4) | 5 (0.8) | 0 (0.0) | 70 (11.2) | 54 (11.2) | 1 (0.3) | 0 (0.0) | 55 (13.9) |
| Vomiting | 38 (6.1) | 8 (1.3) | 0 (0.0) | 46 (7.4) | 29 (7.3) | 1 (0.3) | 0 (0.0) | 30 (7.6) |
| Muscle Pain | 42 (6.7) | 3 (0.5) | 0 (0.0) | 45 (7.2) | 15 (3.8) | 2 (0.6) | 0 (0.0) | 17 (4.3) |
| Fatigue | 28 (4.5) | 7 (1.1) | 0 (0.0) | 35 (5.6) | 27 (6.8) | 1 (0.3) | 0 (0.0) | 28 (7.1) |
| Nausea | 30 (4.8) | 7 (1.1) | 0 (0.0) | 37 (5.9) | 39 (9.8) | 1 (0.3) | 0 (0.0) | 40 (10.1) |
| Abdominal Pain | 28 (4.5) | 3 (0.5) | 0 (0.0) | 31 (5.0) | 19 (4.8) | 0 (0.0) | 0 (0.0) | 19 (4.8) |
| Difficulty in Breathing | 1 (0.2) | 0 (0.0) | 0 (0.0) | 1 (0.2) | 1 (0.3) | 0 (0.0) | 0 (0.0) | 1 (0.3) |
| Cough | 13 (2.1) | 0 (0.0) | 0 (0.0) | 13 (2.1) | 11 (2.8) | 0 (0.0) | 0 (0.0) | 11 (2.8) |
| Joint Pain | 16 (2.6) | 0 (0.0) | 0 (0.0) | 16 (2.6) | 15 (3.8) | 1 (0.3) | 0 (0.0) | 16 (4.0) |
| Muscle Weakness | 13 (2.1) | 0 (0.0) | 0 (0.0) | 13 (2.1) | 14 (3.5) | 1 (0.3) | 0 (0.0) | 15 (3.8) |
| Skin Rash | 0 (0.0) | 1 (0.2) | 0 (0.0) | 1 (0.2) | 1 (0.3) | 0 (0.0) | 0 (0.0) | 1 (0.3) |
| Skin Itching | 3 (0.5) | 1 (0.2) | 0 (0.0) | 4 (0.6) | 3 (0.8) | 0 (0.0) | 0 (0.0) | 3 (0.8) |
| Swelling in Armpit | 1 (0.2) | 0 (0.0) | 0 (0.0) | 1 (0.2) | 0 (0.0) | 0 (0.0) | 0 (0.0) | 0 (0.0) |
| Pain in Armpit | 1 (0.2) | 0 (0.0) | 0 (0.0) | 1 (0.2) | 0 (0.0) | 0 (0.0) | 0 (0.0) | 0 (0.0) |
| Swelling in Groin | 1 (0.2) | 0 (0.0) | 0 (0.0) | 1 (0.2) | 1 (0.3) | 0 (0.0) | 0 (0.0) | 1 (0.3) |
| Pain in Groin | 0 (0.0) | 0 (0.0) | 0 (0.0) | 0 (0.0) | 1 (0.3) | 0 (0.0) | 0 (0.0) | 1 (0.3) |
| Pain in Scrotum | 0 (0.0) | 1 (0.2) | 0 (0.0) | 1 (0.2) | 1 (0.3) | 0 (0.0) | 0 (0.0) | 1 (0.3) |
| Diarrhoea | 19 (3.0) | 1 (0.2) | 1 (0.2) | 21 (3.4) | 11 (2.8) | 1 (0.3) | 0 (0.0) | 12 (3.) |
| Unusual Swelling | 4 (0.6) | 1 (0.2) | 0 (0.0) | 5 (0.8) | 4 (1.0) | 0 (0.0) | 0 (0.0) | 4 (1.0) |
| Overall | 559 (89.7) | 63 (10.1) | 1 (0.2) | 623 | 384(97.0) | 12 (3.0) | 0 (0.0) | 396 |

*This table includes all AEs. Some participants have more than 1 event of the same symptom. For those participants who had the same AE over multiple days, only the highest grade was reported. Symptoms reported according to CTCAE medical coding. The percentage shown is the total number of given type of AE with a given grade and overall, out of total number of AE in the respective treatment.*

*Values reported as n (%). % corresponds to the % of the total AE for that grade. There was only one grade 3 event, diarrhoea in a participant who received*
