# Supplementary material for: An open label, block randomized, community study of the safety and efficacy of co-administered ivermectin, diethylcarbamazine plus albendazole vs. diethylcarbamazine plus albendazole for lymphatic filariasis in India
Source: PLoS Negl Trop Dis. 2021 Feb 16;15(2):e0009069. doi: 10.1371/journal.pntd.0009069 (PMC7909694; doi:10.1371/journal.pntd.0009069)
Supplement: S3 Table — (DOCX) [file pntd.0009069.s004.docx]

**S3 Table.**  Results of univariate and multivariate analysis of the association of CFA clearance with drug regimen, FTS score at baseline and demographic variables

| Factors | No. CFA (+) at baseline | No. (%) CFA (-) 1-year post MDA | Univariate analysis | | Multivariate analysis | |
| --- | --- | --- | --- | --- | --- | --- |
|  |  |  | OR (95% CI) | P value | OR (95% CI) | P value |
| **MDA:** |  |  |  |  |  |  |
| DA | 864 | 124 (14.4) | 1.0 |  | 1.0 |  |
| IDA | 1077 | 108 (10.0) | 0.67 (0.50 - 0.98) | 0.003 | 0.79 (0.48 - 1.30) | 0.355 |
| **Gender:** |  |  |  |  |  |  |
| Female | 918 | 92 (10.0) | 1.0 |  | 1.0 |  |
| Male | 1023 | 140 (13.7) | 1.42 (1.07 - 1.89) | 0.014 | 1.50 (1.11 - 2.01) | 0.008 |
| **Age-class:** |  |  |  |  |  |  |
| Child | 492 | 74 (15.0) | 1.0 |  | 1.0 |  |
| Adult | 1449 | 158 (10.9) | 0.29 (0.22 - 0.38) | p<0.0001 | 0.71 (0.52 - 0.98) | 0.039 |
| **MDA × FTS score at baseline:** |  |  |  |  |  |  |
| DA/IDA: FTS score=1 - weak positive | 283 | 102 (36.0) | 1.0 |  | 1.0 |  |
| DA: FTS score=2 - medium positive | 208 | 33 (15.9) | 0.33 (0.21-0.53) | p<0.0001 | 0.28 (0.16 - 0.48) | p<0.0001 |
| DA: FTS score=3 - strong positive | 536 | 46 (8.6) | 0.17 (0.11 - 0.25) | p<0.0001 | 0.14 (0.08 - 0.23) | p<0.0001 |
| IDA: FTS score=2 - medium positive | 274 | 29 (10.6) | 0.21 (0.13 - 0.34) | p<0.0001 | 0.24 (0.14 - 0.39) | p<0.0001 |
| IDA: FTS score=3 - strong positive | 640 | 24 (3.8) | 0.07 (0.04 - 0.11) | p<0.0001 | 0.65 (0.41 - 1.03) | p<0.0001 |

*OR-Odds ratio*

*CI-Confidence interval*
