# Supplementary figures and images for: An open label, block randomized, community study of the safety and efficacy of co-administered ivermectin, diethylcarbamazine plus albendazole vs. diethylcarbamazine plus albendazole for lymphatic filariasis in India
Source: PLoS Negl Trop Dis. 2021 Feb 16;15(2):e0009069. doi: 10.1371/journal.pntd.0009069 (PMC7909694; doi:10.1371/journal.pntd.0009069)

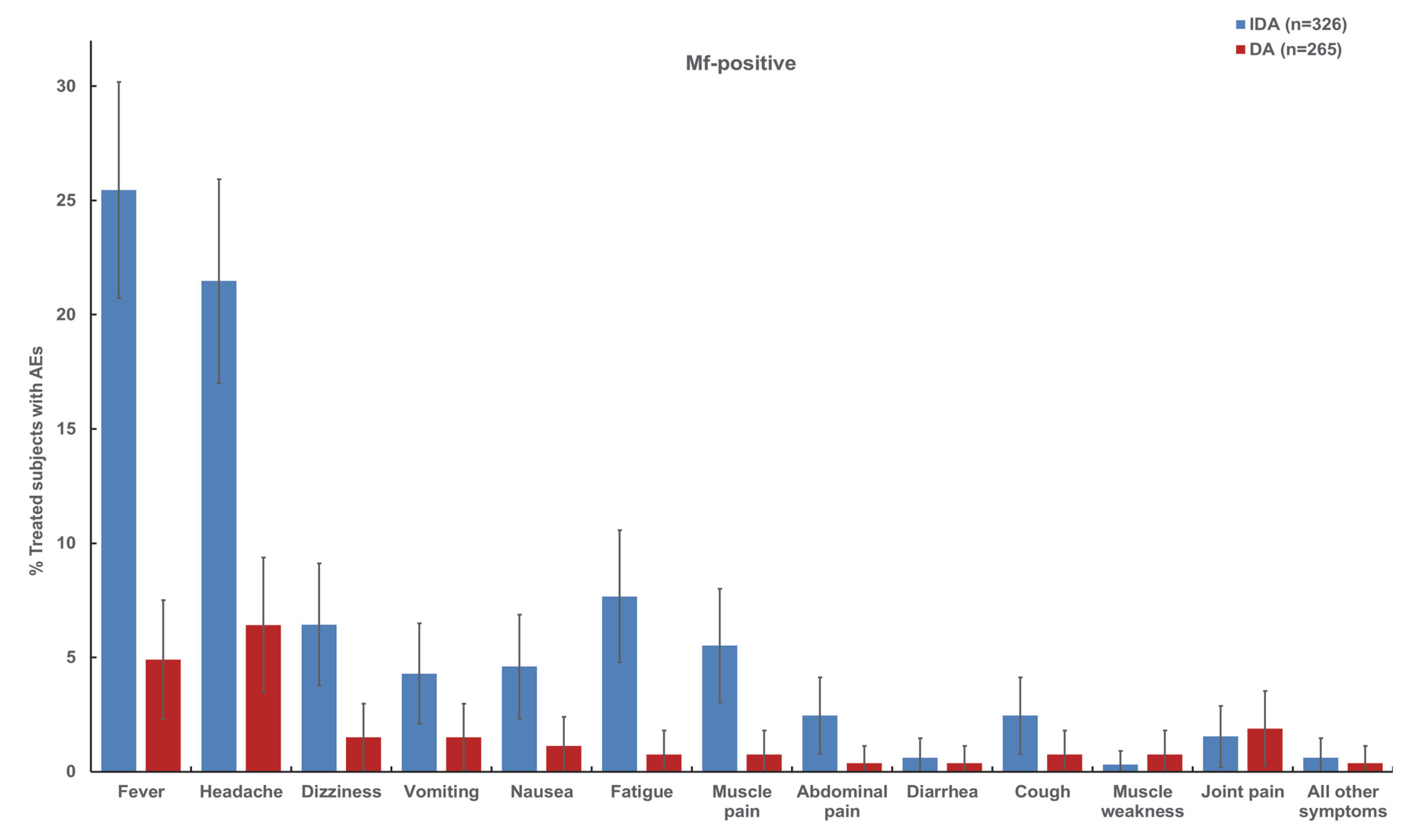

Supplement: S1 Fig — (TIF) [file pntd.0009069.s005.tif]

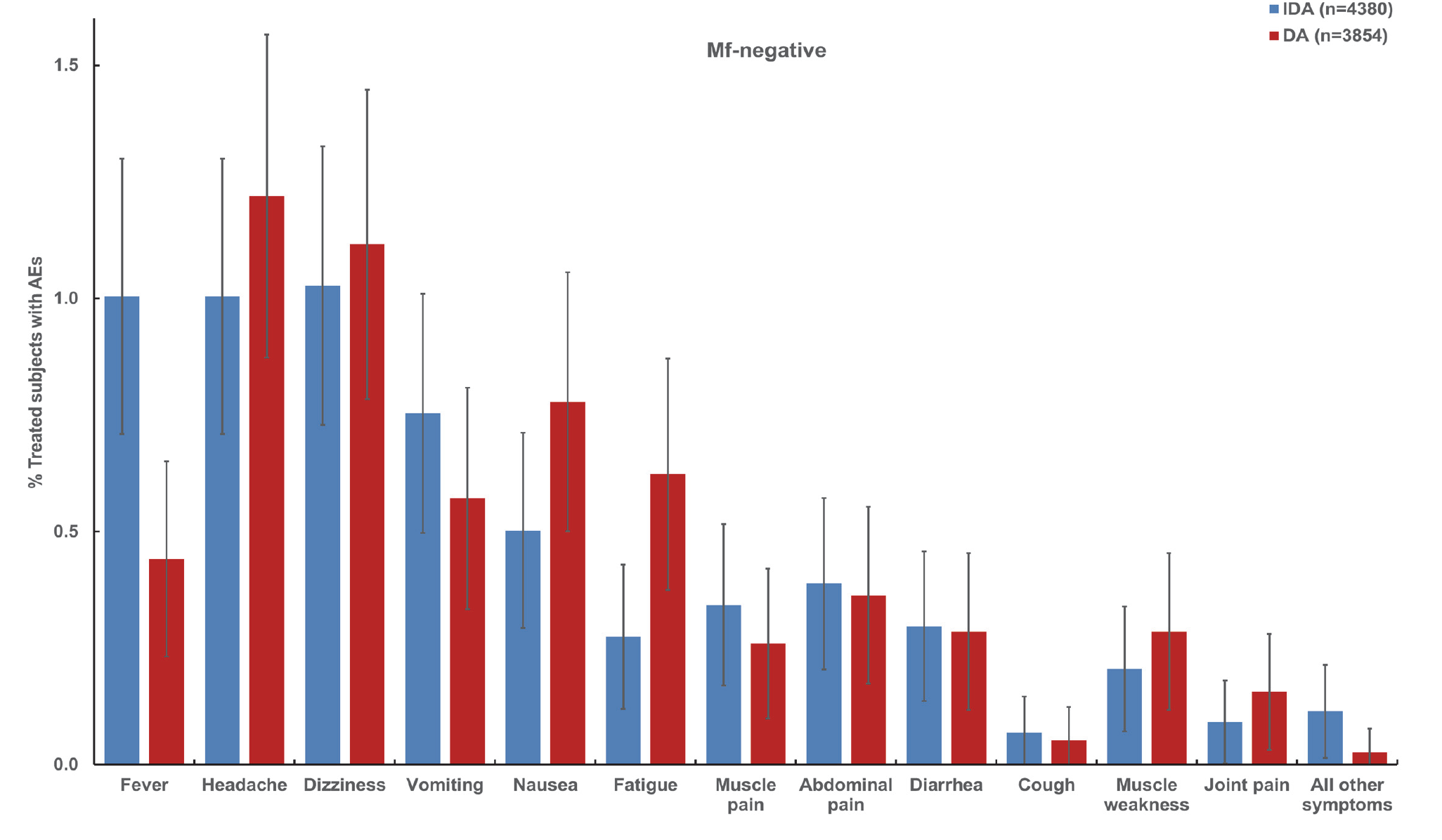

Supplement: S2 Fig — (TIF) [file pntd.0009069.s006.tif]
