# Supplementary material for: An open label, block randomized, community study of the safety and efficacy of co-administered ivermectin, diethylcarbamazine plus albendazole vs. diethylcarbamazine plus albendazole for lymphatic filariasis in India
Source: PLoS Negl Trop Dis. 2021 Feb 16;15(2):e0009069. doi: 10.1371/journal.pntd.0009069 (PMC7909694; doi:10.1371/journal.pntd.0009069)
Supplement: Attachment — Submitted filename: Responses to reviewers comments.docx [file pntd.0009069.s007.docx]

**NB: Our replies are in green and bold. Line nos. are added with reference to the revised main text.**

**Reviewer's Responses to Questions**

Key Review Criteria Required for Acceptance?

As you describe the new analyses required for acceptance, please consider the following:

**Methods**

-Are the objectives of the study clearly articulated with a clear testable hypothesis stated?

-Is the study design appropriate to address the stated objectives?

-Is the population clearly described and appropriate for the hypothesis being tested?

-Is the sample size sufficient to ensure adequate power to address the hypothesis being tested?

-Were correct statistical analysis used to support conclusions?

-Are there concerns about ethical or regulatory requirements being met?

**Reviewer #1:**

The objectives of the study are very clearly articulated, and the study design is appropriate for these objectives. The study population is clearly described, and appropriated. The sample size is adequately powers, and the statistical analyses used are appropriate. I have no concerns about meeting ethical or regulatory requirements.

**Thanks for the positive comments.**

**Reviewer #2:** Yes to above.  Please address the following points:

Line 146:  How were the villages split into the two blocks?  Why 4 villages in one block and 2 in the other?

**A prevalence survey tested convenience samples (n=7589) of residents of 13 villages and one ward in urban area in Yadgir district prior to the study. Those results were used to identify two blocks of villages with comparable infection prevalence and population. Treatment assignment was random but the two villages randomly allocated to DA were larger in population size so only two DA villages were included in the study. The details are included in the revised text (Lines 144-149).**

Lines 170-171:  How were these assumptions determined?

**Randomized clinical trial results showed >90% reduction in Mf-positivity 1-year post-treatment with IDA (Thomson et al, 2016) and >60% reduction 2-year post treatment with DA** (**Bockarie, 2007). This assumption was used and with 80% power to detect 30 % effect size a minimum sample size of 35 was determined. These references are cited in the revised MS (Lines 173-176).**

Line 175:  For readers who are not familiar with India, it would be helpful to describe what anganwadi means.

**Anganwadi Centre is a child care unit for children below 5 years, located for every 800-1000 population. The Centre is run by government of India as part of the Integrated Child Development Services program to combat child hunger and malnutrition. The description is added in the revised MS (Lines 180-182).**

FTS:  Was testing done during household visits, or in a field lab?

**FTS test was done during household visits. This is mentioned in the revised text (Line 222).**

Line 296:  This sentence needs more explanation: “The EDC system is 21 CFR Part 11 compliant”

**Title 21 CFR Part 11 is the part of Title 21 of the Code of Federal Regulations that establishes the United States Food and Drug Administration (FDA) regulations on electronic records and electronic signatures (ERES). Part 11, as it is commonly called, defines the criteria under which electronic records and electronic signatures are considered trustworthy, reliable, and equivalent to paper records. (**[**https://www.fda.gov/regulatory-information/search-fda-guidance-documents/part-11-electronic-records-electronic-signatures-scope-and-application accessed 27 August 2020**](https://www.fda.gov/regulatory-information/search-fda-guidance-documents/part-11-electronic-records-electronic-signatures-scope-and-application%20accessed%2027%20August%202020)**) (Lines 305-311).**

Age groups: The methods described analysis by two age groups - child (<18) or adults.  Was it possible to conduct analyses by smaller age groups?  E.g. Paper by Dubray et al used age 5-9, 10-17, and 18+. <https://journals.plos.org/plosntds/article?id=10.1371/journal.pntd.0008298>

**The data are reported with separate results for adults and minor children. Complete study data will be shared so that interested readers can perform additional analyses.**

**Reviewer #3:** -

Are the objectives of the study clearly articulated with a clear testable hypothesis stated? Yes

-Is the study design appropriate to address the stated objectives? Yes

-Is the population clearly described and appropriate for the hypothesis being tested? Yes

-Is the sample size sufficient to ensure adequate power to address the hypothesis being tested? Yes

-Were correct statistical analysis used to support conclusions? Yes

-Are there concerns about ethical or regulatory requirements being met? No

**Results**

-Does the analysis presented match the analysis plan?

-Are the results clearly and completely presented?

-Are the figures (Tables, Images) of sufficient quality for clarity?

**Reviewer #1:**

The results are very clearly and comprehensively presented. The analyses presented in the Results section match the plan described in the Methods section. The tables and images are generally of sufficient clarity, except for Figure 3 (Forest plot), in which the font size is too small for easy reading.

**Thanks for the positive comments. The font size of Forest plot (Figure 3) has been increased as suggested (Revised Figure 3).**

**Reviewer #2:**

yes, please address the following points:

How many households were visited in total?  Average/range of number of participants per household?

**1652 (77.1%) of the 2149 households in the IDA arm and 1260 (86.4%) of 1459 households in the DA arm were visited. The average (range) no. of participants per household were 4.6 (1-21) and 4.7 (1-20) in the respective arms. The purpose of the study was to enroll participants for treatment and the EDC system was not programmed for entry of households visited but not enrolled.**

Table 2: Results by age groups should be included

**Filarial infection parameters are reported by two broad age groups (children 5-17 years and adults ≥ 18 years) in Table 3.**

Table 3: Results are shown by three age groups here (different to what was described in Methods and the text lines 372).  The first two age groups overlap (6-8 vs 5-17), which is a bit confusing.  Why were results for 6-8 year olds reported separately in this table but not done anywhere else?

**As stated above,** **the data are reported with separate results for adults and children ages 5-17 (Table 3). Complete study data will be shared so that interested readers can perform additional analyses. The results of 6-8-year-old data are removed from Table 3 and in the main text to maintain uniformity in the presentation of age-related results.**

Lines 379-380: Please point the reader to the results, and briefly describe them here.  i.e. what % were Grade 1 and 2 for each arm.

**Agreed. The percentage of Grade 1, 2 and 3 AEs in each arm is given in the revised manuscript (Lines 410-414)**

Table 5 is described in the text before Table 4.

**The statement referring table 4 is brought to the beginning of the paragraph before Table 5 (Lines 417-419)**

Lines 384-387: Briefly describe key results here, including numbers.

**Key results are reported in the text. We do not agree that details such as numbers in the tables should be repeated in the text.**

Figures 4:  please add 95% CI error bars.

**95% CI error bars are added in the revised Figure 4 as suggested**

Figure S1: please add 95% CI error bars.  If the main focus of this study is to compare DA with IDA, it would be better to have IDA vs DA for Mf positives in one panel, and IDA vs DA for Mf negatives in another panel.

**Agreed and 95% CI error bars are added in the revised Figure S1 as suggested. The AEs after IDA vs DA comparison is provided in Fig 4. Figure S1 compares rates for different AEs by Mf status. As suggested, Figure S1 has been revised providing separate panels for Mf positives and Mf negatives.**

Line 424-426:  Some numbers here for the key results would be helpful.  Was the difference in fever significant between IDA and DA?

**Figure 4 provides the details of different types of AE and the percentage of treated persons with fever and headache. However, required data are also given in the revised text for easy reference to the readers as suggested. The difference in fever was significant between IDA and DA (P=.0002) (Figure 4 is now given with error bar) (Lines:484-485).**

Table S2:  There is an error in the partitioning of the first line of the table.

**Thanks for pointing out the formatting error. Error corrected in Table S2 in the revised ms.**

Lines 438-442:  Some numbers here would be helpful.

**There are many columns and extensive data in Fig 4. The text summarizes the results here and details are provided in Fig 4. We do not prefer that data in Fig 4 to be repeated in the text.**

Line 444:  How many developed these reactions?  How long did they last for?  Did any last for longer than the 7 day follow up period?

**Six and two individuals developed localized reactions after IDA and DA respectively. No reactions lasted more than 10 days.** **These details are given in the revised manuscript. (Line: 504-506): The details on the status during follow-up are available in our earlier publication cited (Ref # 21, Kuttiatt et al., 2019).**

Figure 6: How does this compare to the age/sex distribution of those who were still Mf+ after 1 year?

**The question is unclear. The figure clearly shows that Mf clearance rates were high in both treatment groups when baseline Mf counts were low. IDA was significantly more effective than DA for clearing Mf in persons with baseline Mf counts greater than or equal to 32.**

Line 488:  Some may not consider this a “small” difference.

**Agree. Deleted the word “small” (Line: 550).**

Fig 7:  I don’t think this Figure is necessary – results were the same for all groups and can just be described in text.

**Agreed. Deleted Figure 7 and the results are described in the text (Lines: 567-572).**

**Reviewer #3:**

-Does the analysis presented match the analysis plan? Yes-Are the results clearly and completely presented? Yes

-Are the figures (Tables, Images) of sufficient quality for clarity? Yes

**Reviewer #1:**

The conclusions are well supported by the results that are presented, and the limitations are discussed comprehensively. The public health relevance is very clearly explained by the the authors discussion of how the data presented in this paper leads us to a greater understanding of the adverse events and efficacy of using IDA as a tool for the elimination of LF.

**Thanks for the positive comments**

**Reviewer #2**:

yes.  Please address the following points:

The discussion is very long and some sections are repetitive.  Could be made more concise.

**Agreed. The discussion is revised and the repetitive sections removed.**

Line 526:  What were the rates at the other sites?

**AE rates overall and for each of the five study sites are reported in Ref 13 (Weil, GJ et al., 2019) and are not included to avoid repetitions. Also, not considered to provide too much data which are already available in the global safety paper. However, overall AE rates in the global safety study after IDA and DA (12% each) are given in the revised text (Line: 592).**

Line 558: reference needed.

**Reference #23 (Dubray et al., 2020) is cited here (Line: 629).**

Line 576: reference needed.

**Response: Reference # 6 (King et al., 2018) is given (Line: 646).**

Lines 587-588:  Belongs in results

**Agreed. Lines 587 through 591 have been shifted to results (Lines 562-566). The last sentence in the paragraph (lines 591-593) is deleted considering the small number of CFA positive children at baseline who became positive for MF.**

Lines 606-609: Although true, this is not really relevant to the current study, and could be removed.

**Agreed. The text removed in the revised Ms.**

Although IDA was more effective than DA for clearing Mf at one year, 16% of those who received IDA were still Mf positive (and therefore infectious) after one year.  Was IDA expected to be more effective than this?

**Mf prevalence 12 months after IDA in persons with Mf at baseline was lower in this study than in a clinical trial performed in PNG (Ref 6: King et al., 2018) but higher than that reported in a clinical trial performed in the Ivory Coast (Ref 26: Bjerum, et al., 2019). It is possible that some participants were reinfected after IDA treatment. Results may also have been affected by India’s age-based dosing of DEC that provides lower than recommended doses of DEC to larger persons (Goss CW, O’Brian K, Dubray C, Fischer PU, Hardy M, Jambulingam P, et al. (2019) Dosing pole recommendations for lymphatic filariasis elimination: A height-weight quantile regression modeling approach. PLoS Negl Trop Dis 13(7): e0007541. https://doi.org/10.1371/journal. pntd.0007541) (Lines-661-667)**

Similarly, DA was only 61.8% effective at clearing Mf at one year – based on many years of experience with DA, was this result expected?

**This rate was expected. Clearance rates in clinical trials are often lower than this, because trials require high baseline Mf counts for inclusion and usually use membrane filtration to detect Mf.**

Considering that neither DA or IDA reduced CFA significantly after one year, some comments about the use of CFA in post-MDA surveillance would be helpful.

**A recent publication addressed this issue (**[**https://pubmed.ncbi.nlm.nih.gov/32402169/**](https://pubmed.ncbi.nlm.nih.gov/32402169/)**), but it is beyond the scope of this study (King CL, Weil GJ, Kazura JW. Single dose triple drug therapy completely suppresses *Wuchereria bancrofti* microfilaremia for 5 years. N Eng J Med 2020;14:1956-1957). The text has been revised incorporating the results of this study and the reference is cited in the revised Ms (Ref # 25;Lines 654-659)**

**Reviewer #3:**

-Are the conclusions supported by the data presented? Yes

-Are the limitations of analysis clearly described? Yes

-Do the authors discuss how these data can be helpful to advance our understanding of the topic under study? Yes

-Is public health relevance addressed? Yes

**Thanks for the positive comments.**

**Editorial and Data Presentation Modifications?**

**Reviewer #1:**

A few suggestions are made for improvement of this manuscript:

1. In the Methods section, subsection on Study Location (p 7), please provide some data on MDA coverage in the relevant LF MDA Implementation Unit, in the round(s) of MDA prior to initiation of the study. This would provide the relevant context for the statement made in the Discussion that "The study was carried out in an area where compliance for routine MDA for elimination of LF was very low" (lines 623 - 624).

**As suggested, data on MDA coverage is given in the revised manuscript. MDA started in 2004 in Yadgir, completed fifteen annual rounds by 2018 and in 2019, triple drug regimen was introduced.** **Independent coverage surveys conducted in Yadgir after 8,11 and 15 rounds of MDA in 2011, 2015 and 2018 showed consumption rates of 90.1%, 56.2% and 75.4% out of eligible population. The following references are cited (Ref # 14-16; Lines 140-143 & Lines: 684-689).**

2. In the Methods section, subsection on Testing for CFA and Mf, it is stated that “CFA-positive participants were screened for Mf after 9.00 PM by night blood smear examination” (line 220). This implies that only CFA positive participants were screened for Mf by NBS. However, data provided in Table 3 suggests that almost all who were tested for CFA (total of 4732) were also screened for Mf (4706). This discrepancy should be clarified.

**Thanks for pointing out the problem in Table 3. Persons who were tested negative for CFA were assumed to be Mf negative, as CFA is an indication of live adult worm, in the absence of which no mf can be expected. In the table the column showing the number screened for Mf has been appropriately modified. A foot note is given on the denominator used for calculating Mf prevalence. The denominator is all the CFA negative plus number of CFA positive persons screened for Mf. CFA positive persons not tested for Mf are not included (Table 3 footnote).**

3. In the Methods section, subsection on Clinical monitoring and assessment of adverse events, it is stated that "Adverse events were recorded as mild (grade 1), moderate (grade 2) and severe (grade 3)" (lines 256 – 257). However, Table 5 includes an additional grade (Grade 4 AE). Please clarify. Grade 4 means life threatening. No grade 4 AEs occurred in this study.

**Agreed. Grade 4 is included in the methodology (Lines:264). It is mentioned in the results section that no case of Grade 4 occurred in our study (Line: 413-414).**

4. The CONSORT diagram in Figure 2 indicates that non-compliance was much higher in the IDA arm (n=1989) than in the DA arm (n=860). As a proportion of the eligible population in the two arms, this is approximately 30% vs 17%. Some comment on the reasons for this difference in the discussion might be useful for guiding future strategies for the adoption of IDA for LF elimination in India.

**Thanks for pointing out the error in the CONSORT Figure 2. We have checked the raw data and found errors in the reported data. The figure is redrawn after entering the correct values.**

**The proportion of treated among eligible were 73.5% in IDA and 82.9% in DA. The number of persons who were not treated were due to non-compliance. We did not revisit the houses for absentees. This is discussed in the revised manuscript (Lines: 684--693).**

**Reviewer #2:**

Please see above comments regarding figures.

**Figures related to the above comments are well taken and are revised.**

**Reviewer #3:** **(No Response)**

**Reviewer #1:**

This is a very well-written paper that provides detailed information from one study site in a multi-centre study that assessed IDA as a tool in the global programme for the elimination of lymphatic filariasis. The information provided here on adverse events associated with use of IDA and its efficacy in clearance of Mf and CFA 12 months after treatment, is a valuable addition to the evidence base for adoption of this important new tool.

**Thanks for the compliments.**

**Reviewer #2:**

This is an important study about adverse reactions and efficacy of 2-drug vs 3-drug MDA in India.  The study is well designed, and the paper is well written.  The results will contribute significantly to LF elimination efforts.  The paper is suitable for publication in PLOS NTDs after the following points have been addressed.

**Thanks for the compliments.**

**Abstract**

Line 35: Not all AEs were mild, please include %s.

**%s included in the revised text: “Most adverse events (AE) were mild after both treatments; fewer than 0.1% of participants experienced AEs with severity greater than grade 1, and no serious AEs were observed (Lines: 35-37).**

Line 37:  was this difference significant?

**As noted in Table 5, crude AE rates were significantly higher after IDA than after DA (8.3% vs 6.4%, P < 0.01), and we have added the P value to the Abstract (Line: 38). However, AE rates were not significantly different after correction for age and gender. Difficult to present this level of detail in the abstract given word number restrictions.**

**Introduction:**

Lines 72 and 76: different numbers of LF endemic countries (72 vs 73)?

**Agreed. it is typographical error. Corrected as “72 countries are endemic for LF” (Line: 77)**

Line 95:  Why were some districts “hard core”?  Poor coverage, other reasons?

**The National Vector-Borne Diseases Control Programme (NVBDCP) identified 31 districts as “hard core” districts, which have been reporting >1% Mf prevalence persistently, despite several rounds of MDA (**[**https://nvbdcp.gov.in/WriteReadData/l892s/National-Roadmap-ELF.pdf**](https://nvbdcp.gov.in/WriteReadData/l892s/National-Roadmap-ELF.pdf)**). There could be multiple reasons for persistence: high baseline prevalence, sub-optimal MDA coverage / consumption, sub-optimal impact, migration from endemic districts. However, this is beyond the scope of this paper.**

Line 103: Would be helpful to mention where this larger trial was done, and on how many people.

**This trial was done in Papua New Guinea (PNG). 60 people with LF were treated. This has been added in the revised manuscript (Lines: 104-105).**

Line 112-114:  As mentioned later in the manuscript, WHO started recommending large-scale use of IDA from 2017, and countries started implementing this in 2018.

**Yes. This recommendation is cited as Reference 3 in lines 169-170 and it is discussed further in the last paragraph of the text.**

**Reviewer #3:**

The manuscript is one of the major studies that the authors have carried out using the triple drug therapy (Ivermectin/DEC/Albendazole), in continuation of previous studies in other places.

The manuscript is excessively long (but understandably) and might benefit from removing redundant text, that is also present in the detailed supplementary text. No major critiques. This is a massive effort and commendable for the efforts put in. The numbers have been a bit confusing though. Although it might be difficult to compile, a single excel sheet with all of the data could be more useful.

**Comments:**

1. The methods section could be greatly trimmed, as most of it is also included in the supplementary text.

**Repetition of text from the Protocol (Supplementary) in the manuscript text is not a problem. We were required to provide the full protocol as supplementary material. Most readers will not read the protocol, so the manuscript needs to describe methods in sufficient detail so that the paper can stand alone without requiring readers to refer to supplementary material.**

2. Ln 354. 70.1 % received treatment with IDA and 74.9 % received DA. Going to the consort diagram, we see that 4758 of 6475 individuals received IDA (prior to AE assessment). This amounts to 73.48% of eligible individuals. Likewise, 4160 of 5020 individuals received DA (prior to AE assessment), that amounts to 82.86%. Even when the numbers of the ‘Full analysis set included’ are taken, the percentages are 72.2 % and 82.19 %.

**The reviewer has correctly pointed out the error in percentages. The percentages were recalculated and the percentages as given by the reviewer (73.48% for IDA and 82.86% for DA) was found to be correct. The corrected values are given in the revised ms (Lines:372-374)**

3. Further, while the CONSORT diagram excludes 2 individuals each in the IDA and DA arms, the corresponding histogram (Figure 4) with all the AE’s does not exclude them.

Again, in the same CONSORT diagram, doing the math for the excluded individuals (in the box to the top right), 6785 – 38 = 6741 for IDA; so not sure where the 6475 comes from. So assuming 6475 is the right number, excluding non-compliant in IDA (n=1989), we should get 4486 (6475 – 1989 = 4486) instead of 4758. The discrepancies need to be sorted out.

**Thanks for pointing out the error in the CONSORT Figure 2. We have checked the raw data and found errors in the reported data. The figure is redrawn after entering the correct values.**

4. Table 2. 326 (6.9%) in IDA and 265 (6.4%) in DA arm were MF positive before treatment. However, the CONSORT figure (Figure 5), has them as 324 and 264.

**This includes 2 cases in each arm where age and gender details were not available. A footnote is added to indicate this difference in Table 7.**

5. Similarly, the number of people screened for MF were 4706 (IDA) and 4119 (DA), for a total of 8825 individuals. However, the CONSORT figure (Figure 5) states that the number of people tested for MF prior to treatment were 8805.

**The no. tested at baseline for CFA were 8914. Of them 89 were missed or refused for testing Mf leading to a total of 8825. Of these 20 were with “indeterminate” FTS results and for the remaining both FTS and Mf results available. The CONSORT figure (Figure 5) is now revised giving more details on individuals with filarial infections at baseline and who were retested and re-treated 1-year post treatment.**

6. Figure 4. Footnotes state “The overall AE rates or severity of AE differ by treatment regimens (p<0.01) for all comparisons”. If this is true, then all the comparisons made should be significant across the study?

**We believe the reviewer refers table 4. The mistake is corrected in the foot note as “The overall AE rates and severity of AE differ by treatment regimens (P<0.01 for all comparisons)”**

7. Not sure if Figures 6 & 7 adds anything to what is already described in the text and tables and can be removed.

**Agreed for deleting fig. 7. However, Fig 6 can be retained as it illustrates important findings.**

8. Tables 5 & 6 – The last two columns can be deleted. A simple sentence that no AE’s beyond grade 3 were observed.

**Agreed. Removed the last column in table 5 and last two columns in table 6 as suggested.**

9. Most tables have ‘excluded for lack of data for either AE’s, MF or CFA’. Maybe a column can be added to the table(s) to include the number that were excluded, so that the numbers across all the tables/figures match up and easy to interpret.

**We cannot report data that we do not have. We believe that readers will understand this and accept the data as presented. Simple addition shows that very few people were not evaluated for AEs (table 6) and that very few people positive for CFA were not tested for Mf (Table 3).**

10. One of the objectives of the study was “To assess and compare the prevalence of antibody with that of Ag and MF” (Supplementary information). However, I do not seem to find any findings related to this objective.

**Antibody testing was included in the protocol but this was not needed for the safety or efficacy analysis and such testing has not been performed to date.**

11. Will the data be available for the community for any further metanalyses?

**Yes, we will share deidentified data without restriction.**

**********
