# Supplementary material for: An open label, block randomized, community study of the safety and efficacy of co-administered ivermectin, diethylcarbamazine plus albendazole vs. diethylcarbamazine plus albendazole for lymphatic filariasis in India
Source: PLoS Negl Trop Dis. 2021 Feb 16;15(2):e0009069. doi: 10.1371/journal.pntd.0009069 (PMC7909694; doi:10.1371/journal.pntd.0009069)
Supplement: Attachment — Submitted filename: Response to reviewers comments_09 Dec 2020.docx [file pntd.0009069.s008.docx]

**Response to Reviewer's comments (indicated in red)**

Key Review Criteria Required for Acceptance?

As you describe the new analyses required for acceptance, please consider the following:

**Methods**

- Are the objectives of the study clearly articulated with a clear testable hypothesis stated?
- Is the study design appropriate to address the stated objectives?
- Is the population clearly described and appropriate for the hypothesis being tested?
- Is the sample size sufficient to ensure adequate power to address the hypothesis being tested?
- Were correct statistical analysis used to support conclusions?
- Are there concerns about ethical or regulatory requirements being met?

**Reviewer #1: Yes to all of the above.**

**Reviewer #2**

1. Thanks for providing additional details about the sampling strategy:

- Lines 158+: “However, Mf surveys carried out in 2016 (prior to this study), as a part of the programme monitoring and evaluation, showed that the Mf-prevalence was above 1% in in all four sentinel and four spot check sites in the district. In addition to these eight sentinel and spot-check sites, Mf surveys were carried out by our team in five other high-risk villages/wards (sites) prior to inititation of the study.  Mf prevalence in these thirteen sites ranged from 1.1 to 7.1% (total N surveyed, 7,589).”

and

- Line 169+: “The results of Mf-survey data collected prior to the study were used to identify two blocks of 4 and 2 villages that were comparable with respect to Mf-prevalence and population size (Figure 1).”
- However, it is still unclear how the survey villages were selected.  The study design and village selection need to be made more clear so that any biases can be appropriately assessed, particularly when the study has found some unexpected results.

Prior to the study, a total of 13 villages were surveyed for assessing Mf prevalence, jointly by the research team and the district programme to select the study sites. These villages included four sentinel and four spot-check sites (selected by the programme as a part of monitoring and evaluation in the district), and five additional sites identified based on disease prevalence. A cross sectional community survey, with household as the sampling unit was conducted. All the consenting individuals aged above 5 years of age in the systematically selected households were screened for Mf using night blood sample. The sample size was 500 in each village. The results of Mf survey were used to select the study sites. This has been further clarified in the revised manuscript (Please LL 147-156 & 159-162).

2. It’s still not clear why 4+2 villages were chosen (e.g. instead of 3+3 or 5+1).  It seems odd for 4+2 to have been a pre-determined plan.  Or was this decided once the results of the Mf survey (before the start of this study) were available, and the villages already chosen, then split into 4+2 to give approximately equal numbers in each arm?

Yes, this was decided once the results of the Mf survey (before the start of this study) were available. Of the 13 villages, six of villages were identified for the study, considering the logistics and Mf prevalence. These six villages were grouped into two blocks of 4 and 2 villages that were comparable in terms of Mf-prevalence and population size. The blocks were randomized for allocation of the treatment regimens.

3. How were the additional five high-risk villages selected?  Were they purposefully selected because of known previous high prevalence?

Additional five high risk villages were purposefully selected based on disease prevalence. Information on number of clinical cases, collected by the National Programme for LF morbidity management was used.

4. Is it possible to provide more specific details about the Mf prevalence in the sentinel and spot check sites?

Site wise blood survey details are given below:

| Sl. No | PHC | Site | Type of site | No. of blood smears collected | No. positive for Mf | Mf rate (%) | Selected for the study |
| --- | --- | --- | --- | --- | --- | --- | --- |
| 1 | Dornahally CHC | Dornahally Urban | Sentinel | 601 | 27 | 4.49 |  |
| 2 | Konkal | Nazarapura |  | 606 | 36 | 5.94 | Yes |
| 3 | Hasnapura | Rangampet |  | 633 | 13 | 2.05 |  |
| 4 | Petammapura | Petammapura |  | 606 | 10 | 1.65 |  |
| 13 | Yadgir | Yadgir town | Spotcheck | 494 | 16 | 3.24 |  |
| 6 | Hattikunni | Hattikunni |  | 606 | 43 | 7.10 | Yes |
| 5 | Gajarkote | Gajarkote |  | 640 | 38 | 5.94 | Yes |
| 8 | Konkal | Anpur |  | 603 | 39 | 6.47 | Yes |
| 7 | Konkal | Konkal | Additional | 604 | 25 | 4.14 |  |
| 9 | Kandakur | Kandakur |  | 631 | 40 | 6.34 | Yes |
| 12 | Kandakur | Gunjanur |  | 516 | 18 | 3.49 | Yes |
| 10 | Kandakur | Ganapur |  | 522 | 34 | 6.51 |  |
| 11 | Kandakur | Dantapur |  | 527 | 27 | 5.12 |  |
|  |  |  |  | 7589 | 366 | 4.82 |  |

5. Thanks for providing additional information about households in the response, but I can’t see this information in the manuscript: “1652 (77.1%) of the 2149 households in the IDA arm and 1260 (86.4%) of 1459 households in the DA arm were visited. The average (range) no. of participants per household were 4.6 (1-21) and 4.7 (1-20) in the respective arms.”

Analyses should have taken into account the number of participants per household, particularly because clustering of CFA-positive and Mf-positive individuals within households have been identified in LF studies elsewhere.  Considering that there was a large range in the number of household participants (1-21 for IDA and 1-20 for DA), household-level clustering could have significantly affected prevalence estimates and confidence intervals for CFA and Mf. Clustering should therefore be assessed (e.g. using intra-cluster correlation - ICC), and prevalence estimates for CFA and Mf adjusted for household clustering if necessary.  I note that the mixed effect logistic regression model used village (cluster) as a random effect, but did not consider household.  Adverse events might also cluster within households, especially subjective symptoms.

This is a good suggestion. However, the electronic data collection system (EDC), used for this safety and efficacy study was not programmed to collect the household details (house ID and the GPS). Therefore, the data base does not have the household details for analyzing the household-level clustering effect.

**Reviewer #3: (No Response)**

--------------------

**Results**

- Does the analysis presented match the analysis plan?</br>
- Are the results clearly and completely presented?</br>
- Are the figures (Tables, Images) of sufficient quality for clarity?</br></br>

**Reviewer #1: Yes to all of the above.**

6. However, as in Table 3, Table 2 should also indicate that the total numbers given as screened for microfilaraemia include those who were CFA negative, and therefore not subjected to night blood testing

Agreed. Added a footnote in Tables 2 and 3 as suggested “.

7. Moreover, there is some disparity in the numbers presented in Tables 2 and 3 and in Figure 5. Tables 2 and 3 indicate that a total of 8887 persons were screened for CFA (4155 DA + 4732 IDA) whereas the CONSORT diagram in Figure 5 states that 8914 persons were tested for CFA prior to treatment.

The typo error in Figure 5 is corrected. The no. tested at baseline for CFA were 8914. Of them 69 were either missed/ refused for testing Mf and missing FTS results and 20 were with “indeterminate” FTS results, leading to a total of 8825. Consequently, the number tested for Mf were 8825. The CONSORT figure (Figure 5) is now re-revised.

8. Similarly, Table 2 indicates that a total of 8825 persons were screened for mf (4119 DA + 4706 IDA), but Figure 5 indicates that 8805 persons were screened for mf prior to treatment. These discrepancies should be rectified.

Agreed. The discrepancies between Table 2 and Figure 5 is rectified. The number screened for Mf was 8825 as given in Table 2. Figure 5, data were corrected as indicated in point no. 7.

**Reviewer #2:**

9. Yes, but please refer to my comments above under Methods.  Some of the prevalence estimates might need to be amended.

As the household details are not available, the effect of household clustering on prevalence could not be adjusted.

**Reviewer #3: (No Response)**

--------------------

**Conclusions**

- Are the conclusions supported by the data presented?</br>
- Are the limitations of analysis clearly described?</br>
- Do the authors discuss how these data can be helpful to advance our understanding of the topic under study?</br>
- Is public health relevance addressed?</br></br>

**Reviewer #1: Yes to all of the above**

**Reviewer #2: Yes**

**Reviewer #3: (No Response)**

--------------------

**Editorial and Data Presentation Modifications?**

Use this section for editorial suggestions as well as relatively minor modifications of existing data that would enhance clarity. If the only modifications needed are minor and/or editorial, you may wish to recommend “Minor Revision” or “Accept”.

**Reviewer #1: A few typographical errors need correction:**

10. Page 6 of the pdf document, line 4: 'Krishnamoorthy' written as 'Krishnamoorhty'

Corrected as “Krishnamoorthy”

11. Page 12, line 141: 'study' written as 'srudy'

Corrected the typo-error (Line 144)

12. Page 20, line 345: p is missing in 'positive'

Corrected the typo-error (Line 353)

13. Page 25, line 414: missing bracket

Missing bracket is given (Line 424).

14. Page 33, line 567: 'comparable' written as 'comparbable'

Accepted and corrected in the revised MS (Line 577)

15. Figure 6, x-axis label: 'prior' written as 'priror'

Accepted and corrected in Figure 6 of the revised MS

**Reviewer #2:**

16. Typo in line 389 – should be CFA-positive.

Accepted and corrected in the revised MS (Line 353)

**Reviewer #3: (No Response)**

--------------------

**Summary and General Comments**

Use  this section to provide overall comments, discuss strengths/weaknesses of the study, novelty, significance, general execution and scholarship. You may also include additional comments for the author, including concerns about dual publication, research ethics, or publication ethics. If requesting major revision, please articulate the new experiments that are needed.

**Reviewer #1:** This revised manuscript addresses most of my concerns, except for the matter described above

**Reviewer #2:** Many thanks to the authors for providing comprehensive responses to my questions.  Again, this is an important study and will contribute significant knowledge for the Global Programme to Eliminate LF, and I congratulate the authors on the excellent work.  I have two remaining questions regarding methods (as described above) - I believe that it is important to address these questions before acceptance for publication.

**Reviewer #3:** I do not have any major comments. The comments raised earlier appears to have been satisfactorily addressed.

Thanks for the feedback

--------------------

Figure Files:

While revising your submission, please upload your figure files to the Preflight Analysis and Conversion Engine (PACE) digital diagnostic tool, https://pacev2.apexcovantage.com. PACE helps ensure that figures meet PLOS requirements. To use PACE, you must first register as a user. Then, login and navigate to the UPLOAD tab, where you will find detailed instructions on how to use the tool. If you encounter any issues or have any questions when using PACE, please email us at [figures@plos.org.](mailto:figures@plos.org.)

Data Requirements:

Please note that, as a condition of publication, PLOS' data policy requires that you make available all data used to draw the conclusions outlined in your manuscript. Data must be deposited in an appropriate repository, included within the body of the manuscript, or uploaded as supporting information. This includes all numerical values that were used to generate graphs, histograms etc.. For an example see here: <http://www.plosbiology.org/article/info%3Adoi%2F10.1371%2Fjournal.pbio.1001908#s5>.

Reproducibility:

To enhance the reproducibility of your results, PLOS recommends that you deposit laboratory protocols in [protocols.io](http://protocols.io/), where a protocol can be assigned its own identifier (DOI) such that it can be cited independently in the future. For instructions see <http://journals.plos.org/plosntds/s/submission-guidelines#loc-materials-and-methods>

__________________________________________________

In compliance with data protection regulations, you may request that we remove your personal registration details at any time.  (Use the following URL: <https://www.editorialmanager.com/pntd/login.asp?a=r>). Please contact the publication office if you have any questions.
